# Neoantigen-specific cytotoxic Tr1 CD4 T cells suppress cancer immunotherapy

**DOI:** 10.1038/s41586-024-07752-y

**Published:** 2024-07-24

**Authors:** Hussein Sultan, Yoshiko Takeuchi, Jeffrey P. Ward, Naveen Sharma, Tian-Tian Liu, Vladimir Sukhov, Maria Firulyova, Yuang Song, Samuel Ameh, Simone Brioschi, Darya Khantakova, Cora D. Arthur, J. Michael White, Heather Kohlmiller, Andres M. Salazar, Robert Burns, Helio A. Costa, Kelly D. Moynihan, Yik Andy Yeung, Ivana Djuretic, Ton N. Schumacher, Kathleen C. F. Sheehan, Marco Colonna, James P. Allison, Kenneth M. Murphy, Maxim N. Artyomov, Robert D. Schreiber

**Affiliations:** 1 Department of Pathology and Immunology, Washington University School of Medicine, St. Louis, MO USA; 2 The Andrew M. and Jane M. Bursky Center for Human Immunology and Immunotherapy Programs, Washington University School of Medicine, St. Louis, MO USA; 3 Department of Medicine, Division of Oncology, Washington University School of Medicine, St. Louis, MO USA; 4 Department of Immunology, University of Texas MD Anderson Cancer Center, Houston, TX USA; 5 Almazov National Medical Research Centre, St. Petersburg, Russia; 6 Oncovir Inc., Washington, DC USA; 7 Natera Inc., Austin, TX USA; 8 Asher Biotherapeutics, South San Francisco, CA USA; 9 Netherlands Cancer Institute, Oncode Institute, Amsterdam, Leiden University, Leiden, Netherlands; 10 The Parker Institute for Cancer Immunotherapy, San Francisco, CA USA

**Keywords:** Immunosuppression, Cellular immunity, Immunization

## Abstract

CD4^+^ T cells can either enhance or inhibit tumour immunity. Although regulatory T cells have long been known to impede antitumour responses^1–5^, other CD4^+^ T cells have recently been implicated in inhibiting this response^6,7^. Yet, the nature and function of the latter remain unclear. Here, using vaccines containing MHC class I (MHC-I) neoantigens (neoAgs) and different doses of tumour-derived MHC-II neoAgs, we discovered that whereas the inclusion of vaccines with low doses of MHC-II-restricted peptides (LDVax) promoted tumour rejection, vaccines containing high doses of the same MHC-II neoAgs (HDVax) inhibited rejection. Characterization of the inhibitory cells induced by HDVax identified them as type 1 regulatory T (Tr1) cells expressing IL-10, granzyme B, perforin, CCL5 and LILRB4. Tumour-specific Tr1 cells suppressed tumour rejection induced by anti-PD1, LDVax or adoptively transferred tumour-specific effector T cells. Mechanistically, HDVax-induced Tr1 cells selectively killed MHC-II tumour antigen-presenting type 1 conventional dendritic cells (cDC1s), leading to low numbers of cDC1s in tumours. We then documented modalities to overcome this inhibition, specifically via anti-LILRB4 blockade, using a CD8-directed IL-2 mutein, or targeted loss of cDC2/monocytes. Collectively, these data show that cytotoxic Tr1 cells, which maintain peripheral tolerance, also inhibit antitumour responses and thereby function to impede immune control of cancer.

## Abstract (web summary)

Type 1 regulatory T cells (Tr1) represent a major obstacle that compromises naturally occurring and therapeutically induced tumour-specific immunity.

## Main

A desirable outcome of cancer immunotherapy is the generation of tumour-specific CD8^+^ cytolytic T cells (CTLs) capable of destroying tumours. Whereas induction of these effector cells is often promoted by ‘helper’ CD4^+^ T cells^8–13^, this process can be inhibited by CD4^+^FOXP3^+^ regulatory T (T_reg_) cells^1–5^. Recent studies have suggested that conventional T_reg_ cells may not be the only immunosuppressive CD4^+^ T cell population residing in progressively growing tumours^6,7^, but establishing the origins, nature and function of the latter has been challenging. This has placed theoretical and practical constraints on cancer immunotherapy in general and cancer-specific neoAg vaccines in particular. The ability of CD4^+^ Tr1 cells to maintain tolerance has been well established in autoimmunity and chronic infections^14–16^ and has been suggested to contribute to cancer immunosuppression^17^. However, Tr1 cells have not been defined based on a master transcription factor but rather are identified as FOXP3-negative, IL-10-producing inhibitory cells^18^. Here we use our well-characterized, syngeneic mouse sarcoma models to show that, whereas synthetic long peptide (SLP) neoAg vaccines containing tumour-specific MHC-I neoAgs and low inputs of tumour-specific MHC-II neoAgs efficiently promote tumour rejection, similar vaccines expressing high quantities of the same MHC-II neoAgs unexpectedly induce immunosuppressive CD4^+^ T cells distinct from T_reg_ cells that inhibit tumour elimination. We characterize these cells as cytolytic Tr1 cells, provide insights into their development and mechanisms of action, and validate strategies to circumvent their inhibitory activity. We further show that a similar CD4^+^ T cell population can be found in progressively growing tumours in mice as they become insensitive to anti-PD1 therapy and in human patients with cancer who respond poorly to immune checkpoint therapy (ICT) and cancer vaccines. We demonstrate that eliminating these cells or inhibiting their function renders cancer immunotherapy more effective. This study thus not only documents a new function for a known CD4^+^ T cell subpopulation but also provides a strategy to circumvent their inhibitory effects on tumour rejection.

## Complex MHC-II dosing profiles by peptide vaccines

**Fig. 1:**
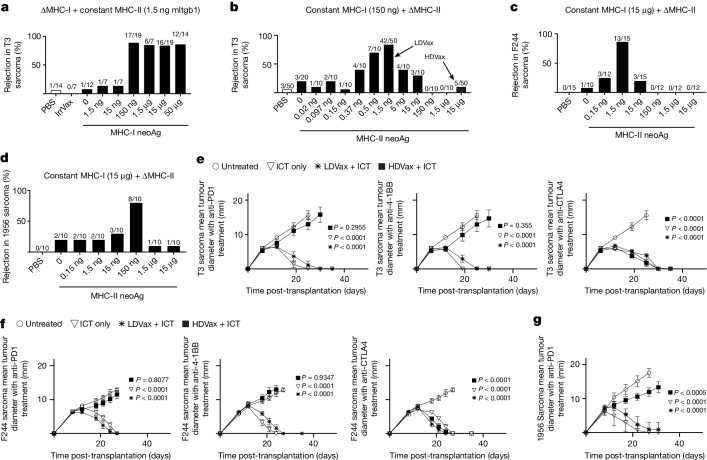
High MHC-II neoAg doses inhibit the antitumour efficacy of SLP vaccines and certain ICTs. **a**, Percent survival of T3 tumour-bearing mice after treatments with vaccines containing fixed (1.5 ng) doses of mItgb1 SLP plus different doses of mLama4/mAlg8 SLP or IrrVax. **b**, Percent survival of T3 tumour-bearing mice after treatments with vaccines containing fixed doses of mLama4/mAlg8 SLP (0.15 μg each) plus different doses of mItgb1 SLP. **c**, Percent survival of F244 tumour-bearing mice after treatment with vaccines containing fixed doses of mPex14 SLP (15 μg per mouse) plus different doses of mPlec SLP. **d**, Percent survival of 1956 tumour-bearing mice after treatment with vaccines containing fixed doses of 1956 mPsmd6 SLP (15 μg per mouse) plus different doses of mCs SLP or IrrVax. The numbers above each bar represent the number of mice that rejected the tumours over the total number of mice used. **e**, T3 tumour outgrowth in mice vaccinated and treated with anti-PD1, anti-41-BB or anti-CTLA4 plus T3-HDVax or T3-LDVax as detailed in the Methods (*n* = 4). **f**, F244 tumour outgrowth in WT mice vaccinated and treated with F244-HDVax or F244-LDVax plus different antibodies (*n* = 4). In panels **e**,**f**, treatment with the three monoclonal antibodies was done in the same experiment using the same cohort of untreated mice as a control. **g**, 1956 Tumour outgrowth in mice vaccinated with 1956-HDVax or 1956-LDVax and treated with anti-PD1 (*n* = 5). Data in panels **e**–**g** represent mean tumour diameter ± s.e.m. and are representative of three independent experiments. *P* values were calculated by comparing the different treatments to untreated control mice and were calculated using two-way analysis of variance (ANOVA), with multiple comparisons corrected using Sidak’s multiple comparison test. Source Data

We previously identified the unique major MHC-I and MHC-II neoAgs expressed in three of our methylcholanthrene (MCA) sarcoma lines (Supplementary Table 1), showed that immune rejection of each tumour depends on CD4^+^ and CD8^+^ T cell responses to the neoAgs uniquely expressed in each tumour line, and further showed that optimal rejection efficacy required the presence of both MHC-I and MHC-II neoAgs in tumours and vaccines^8,19,20^. We were intrigued by the profound effects that CD4^+^ T cells had on CD8^+^ T cell-dependent protective immunity against MCA sarcomas that do not express MHC-II proteins, and therefore decided to study the interdependency between MHC-I and MHC-II neoAg responses in promoting antitumour efficacy. We approached this question by defining the MHC-I and MHC-II neoAg doses either alone or together that induced optimal antitumour efficacy. T3-specific SLP vaccines comprising only T3-specific MHC-I (G1254V mLama4 plus A506T mAlg8) or MHC-II (N710Y mItgb1) SLP plus poly-ICLC adjuvant or irrelevant vaccines containing human papillomavirus (HPV)-SLP (IrrVax) were ineffective in inducing T3 rejection when administered 6 days post-tumour cell inoculation (Fig. 1a,b). However, T3-specific vaccines containing MHC-I neoAgs at doses between 150 ng and 50 μg together with constant low doses of mItgb1 SLP (1.5 ng) induced tumour rejection in nearly all tumour-bearing mice (Fig. 1a,b). By contrast, when doses of T3-specific MHC-I neoAg SLPs were held constant at 150 ng and the dose of T3-specific MHC-II neoAg SLPs was varied, a bell-shaped dose–response curve was observed displaying maximum protection in 42 of 50 vaccinated mice at doses of 1.5 ng of I-A^b^-restricted mItgb1 SLP (hereafter referred to as LDVax; Fig. 1b). Vaccines containing the same dose of T3-specific MHC-I SLP plus higher doses of mItgb1 SLP, for example, 150 ng, induced little or no antitumour efficacy (hereafter referred to as HDVax). Thus, high doses of tumour-specific MHC-II SLP render T3-specific vaccines ineffective.

## Generalizability of HDVax ineffectiveness

To address whether the lack of efficacy of T3-specific HDVax was generalizable to other antigenically distinct tumours, we used vaccines containing the major MHC-I-restricted and MHC-II-restricted neoAgs for two other MCA sarcoma models: F244 (expressing an H2-K^b^-restricted G122A mutation in peroxisomal membrane protein 14 (mPex14) and a D2944Y I-A^b^-restricted mutation in plectin-1 (mPlec)) and 1956 (expressing a H2-K^b^-restricted M50I mutation in proteasome 26S subunit, non-ATPase 6 (mPsmd6) and an I-A^b^-restricted R144S mutation in citrate synthase (mCs))^20^ (Supplementary Table 1). As seen with T3-specific HDVax and T3 tumours, the same type of bell-shape curve for MHC-II neoAgs was observed with F244 and 1956 tumours and their specific HDVax (Fig. 1c,d).

## HDVax inhibits antitumour efficacy of ICT

To discern whether HDVax actively inhibited immunotherapy, we assessed the effect of T3-HDVax on the efficacy of anti-PD1, anti-4-1BB or anti-CTLA4 as each of these antibodies, when administered as monotherapy, induced complete in vivo rejection of T3 tumours^8,19,21^ (Fig. 1e). Combining ICT and LDVax did not alter the antitumour efficacy of the checkpoint antibodies. By contrast, T3-HDVax abrogated tumour rejection in all mice treated with anti-PD1 or anti-4-1BB, but did not ablate anti-CTLA4-induced tumour rejection. Similar results were obtained using the F244 and 1956 tumour models and their respective neoAg vaccines (Fig. 1f,g), demonstrating that HDVax treatment inhibits certain types of ICT. The latter observation was explained by subsequent experiments.

## Antigen specificity of HDVax inhibition

**Fig. 2:**
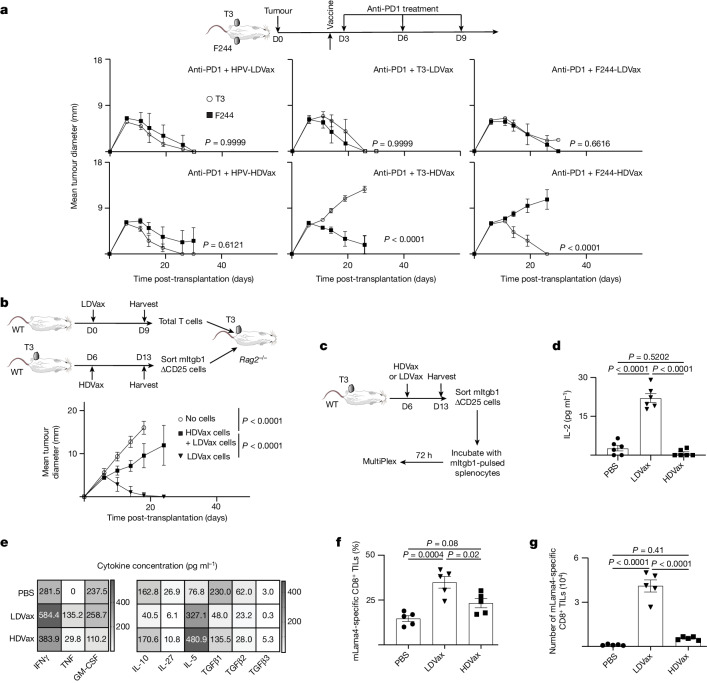
HDVax inhibition is specific for the MHC-II neoAgs in the tumour. **a**, T3 and F244 tumour outgrowth in opposite contralateral flanks of male 129S6 WT mice vaccinated and treated with anti-PD1 (*n* = 5, representative of two experiments). D0, day 0. **b**, T3 tumour outgrowth in *Rag2*^−/−^ mice receiving total T cells (5 × 10^6^) from LDVax-treated mice only or plus CD25-negative mItgb1-specific CD4^+^ T cells (0.5 × 10^6^) sorted from HDVax-treated mice (*n* = 10). Data in panels **a**,**b** are shown as mean tumour diameter ± s.e.m., and statistics were done using two-way ANOVA, with multiple comparisons corrected using Sidak’s multiple comparison test. **c**–**e**, CD4^+^ T cells were purified and stimulated as shown. IL-2 production in the supernatant was assessed using MultiPlex (*n* = 6, pooled from two experiments; **d**). The heatmap displays the production levels of different cytokines (indicated on the *x* axis; **e**). The numbers in panel **e** represent the mean of two independent biological replicates (representative of two experiments). Frequency (**f**) and numbers (**g**) of mLama4^+^-specific CD8^+^ TILs in T3 tumours harvested on day 13 (*n* = 5, representative of three experiments) are also shown. Data in panels **d**,**f**,**g** are expressed as mean ± s.e.m.; statistics were done using one-way ANOVA, with multiple comparisons corrected with Tukey’s method. Source Data

To test the antigen specificity of HDVax inhibition, T3 sarcoma cells and antigenically distinct sex-matched syngeneic F244 sarcoma cells were injected into contralateral flanks of naive 129S6 wild-type (WT) mice. Three days later, mice were treated with anti-PD1 plus either irrelevant HPV-LDVax or HPV-HDVax, T3-LDVax or T3-HDVax, or F244-LDVax or F244-HDVax, and tumour growth was monitored. Neither LDVax nor irrelevant HPV-HDVax treatment inhibited anti-PD1-induced rejection of T3 or F244 tumours (Fig. 2a, upper three panels and lower left panel). However, T3-HDVax treatment selectively inhibited anti-PD1-dependent rejection of T3 but not F244 tumours (Fig. 2a, bottom middle panel). Conversely, F244-HDVax treatment selectively inhibited anti-PD1-dependent rejection of F244 tumours but not T3 tumours (Fig. 2a, bottom right panel). Thus, the inhibitory action of HDVax is specifically linked to the MHC-II neoAg expressed by each tumour line.

## HDVax-induced CD4^+^ T cells inhibit tumour rejection

To formally test whether inhibition was due to induction of immunosuppressive CD4^+^ T cells, we established an adoptive T cell transfer (ACT) system in *Rag2*^−/−^ mice (see Methods). ACT of T_reg_ cell-depleted CD4^+^/CD8^+^ T cells from LDVax-treated WT mice into T3 tumour-bearing, immunodeficient *Rag2*^−/−^ mice was sufficient to induce T3 rejection (Fig. 2b). However, mixing HDVax-induced, T_reg_ cell-depleted mItgb1-specific CD4^+^ T cells with LDVax-induced effector cells abrogated T3 tumour rejection. These data suggested that HDVax induces a CD4^+^ T cell population distinct from classical T_reg_ cells, which possesses potent immunosuppressive activity.

## Characteristics of HDVax-induced CD4^+^ T cells

**Extended Data Fig. 1:**
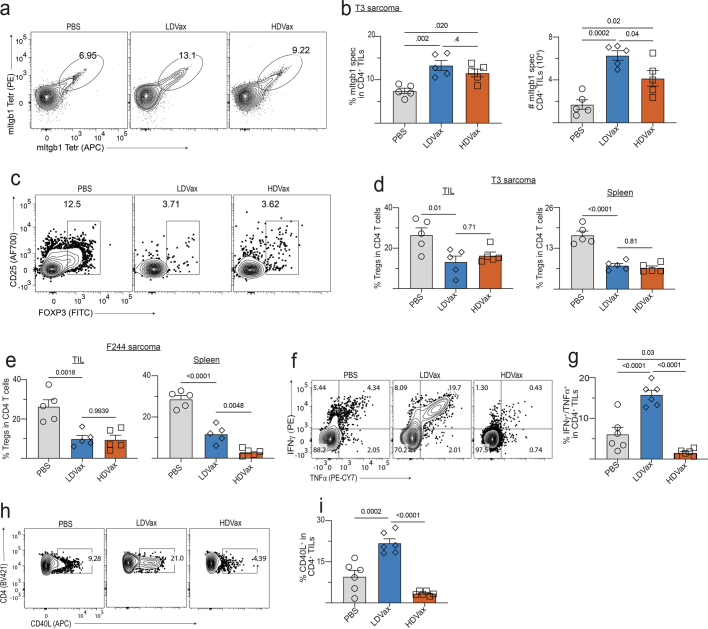
Phenotypic profiles of HDVax versus LDVax-induced CD4^+^ T cells. **a**, representative flow cytometry plots showing mItgb1 tetramer staining in CD4^+^ TILs. **b**, frequency **(left)** and numbers **(right)** of mItgb1-specific CD4^+^ TILs assessed in day 13 T3 tumours (n = 5, representing five experiments). **c**, representative flow cytometry plots showing CD25 and Foxp3 staining in mItgb1-specific CD4^+^ TILs. **d**, frequency of CD25^+^Foxp3^+^ cells in mItgb1-specific CD4^+^ T cells in TILs **(left)** and spleens **(right)** of T3 tumour-bearing mice (n = 5, representing five experiments). **e**, frequency of CD25^+^Foxp3^+^ Tregs in CD4^+^ T cells in tumours **(left)** and spleens **(right)** of F244 tumour-bearing mice (n = 5, representing three experiments). **f**, representative flow cytometry plots showing the frequency of IFNγ/TNFα in CD4^+^ TILs. **g**, frequency of IFN-γ/TNF-α expressing CD4^+^ TILs in multiple mice (n = 6, pooled from two experiments). **h**, representative flow cytometry plots showing CD40L expression in CD4^+^ TILs. **i**, frequency of CD40L expression in CD4^+^ TILs of T3 tumours after in vitro activation (n = 6, pooled from two experiments). Data in all panels are shown as means ± s.e.m. *P values* were calculated using one-way ANOVA with multiple comparisons corrected with Tukey’s method. Source Data

The frequency and absolute numbers of mItgb1-specific CD4^+^ tumour-infiltrating lymphocytes (TILs) from HDVax-treated or LDVax-treated mice were similar (Extended Data Fig. 1a,b). Flow cytometry analyses revealed that the frequency and absolute numbers of T_reg_ cells in mItgb1-specific CD4^+^ TILs or spleens decreased comparably following either HDVax or LDVax treatment compared with PBS-treated controls (Extended Data Fig. 1c,d). Similar findings were observed with F244 tumour-bearing mice treated with F244-HDVax or F244-LDVax (Extended Data Fig. 1e). Cytokine multiplex analyses revealed distinct phenotypes of CD4^+^ TILs induced by LDVax versus HDVax. Whereas T3-LDVax-induced mItgb1-specific CD4^+^ T cells produced IL-2 when stimulated with mItgb1-pulsed splenocytes, T3-HDVax-induced mItgb1-specific CD4^+^ T cells failed to produce IL-2 (Fig. 2c,d). In addition, whereas T3-LDVax-induced CD4^+^ T cells produced high levels of IFNγ, TNF and GM-CSF (Fig. 2e), T3-HDVax-induced CD4^+^ T cells produced high levels of IL-10, TGFβ, IL-5 and IL-27. These results were recapitulated by intracellular staining analyses (Extended Data Fig. 1f,g). Moreover, whereas mItgb1-specific CD4^+^ TILs harvested from T3-LDVax-treated mice expressed increased levels of CD40L following antigen stimulation (LDVax: 22.8 ± 4.6%; PBS: 9 ± 7.4%), only a small fraction of antigen-stimulated T3-HDVax-induced mItgb1-specific CD4^+^ TILs expressed CD40L (HDVax: 3.4 ± 2.0%; Extended Data Fig. 1h,i).

**Extended Data Fig. 2:**
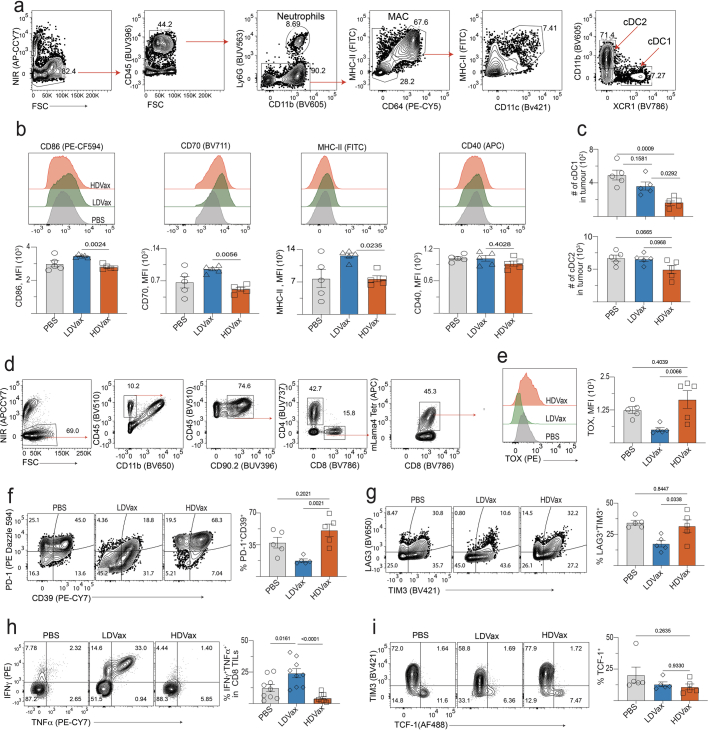
Characterization of dendritic cells and CD8^+^ TILs in HDVax, LDVax and PBS-treated mice. **a**, representative flow cytometry plots showing the gating strategy for cDC1 and cDC2. **b**, expression of CD86, CD70, MHC-II and CD40 on the surface of dendritic cells (CD45^+^CD90.2^−^CD11c^+^MHC-II^+^CD64^−^) in T3 tumours harvested 5 days after vaccination (n = 5, represent two experiments). **c**, numbers of cDC1 **(top)** and cDC2 **(bottom)** in T3 tumours assessed five days post-vaccination (n = 5, represent three experiments). **d**, representative flow cytometry plots showing the gating strategy for mLama4-specific CD8^+^ TILs in day 13 T3 TILs. **e-h**, frequency of mLama4-specific CD8^+^ TILs expressing **e**, TOX **f**, PD-1/CD39 and **g**, TIM-3/LAG3 (n = 5, represent two experiments). **h**, frequency of cells expressing IFN-γ/TNF-α in mLama4^+^ specific CD8^+^ TILs after in vitro activation with splenocytes pulsed with 1μg/ml mLama4 SLP in the presence of Golgi-plug followed by intracellular staining (n = 8, pooled from three experiments). **i**, frequency of mLama4-specific CD8^+^ TILs showing TIM3 and TCF-1. Data in all panels are expressed as means ± s.e.m. *P* values were calculated using one-way ANOVA with multiple comparisons corrected with Tukey’s method. Source Data

Consistent with T3-LDVax-dependent elevated expression of CD40L on CD4^+^ TILs, dendritic cells from the same mice displayed slightly higher but significantly enhanced expression of CD86 and CD70 than comparable cells from T3-HDVax-treated mice. No significant differences were observed in expression levels of CD40 on dendritic cells (Extended Data Fig. 2a,b). Of note, the number of cDC1s was reduced by 2.9-fold in tumours from T3-bearing mice treated with T3-HDVax compared with PBS (Extended Data Fig. 2c). Only a minor reduction in the number of cDC2s was observed, and this was not statistically significant.

In agreement with the aberrant cytokine response and low recovery of cDC1s from T3-HDVax-treated mice, the frequencies and absolute numbers of mLama4-specific CD8^+^ TILs were significantly lower than CD8^+^ TILs harvested from T3-LDVax-treated mice (Fig. 2f,g and Extended Data Fig. 2d). Furthermore, mLama4-specific CD8^+^ TILs from T3-HDVax-treated mice expressed high levels of exhaustion markers (PD1, CD39, TIM3, LAG3 and TOX) and produced low amounts of IFNγ and TNF compared with similar populations from T3-LDVax-treated tumour-bearing mice (Extended Data Fig. 2d–h). No differences were observed in the TIM3^−^TCF1^+^ subpopulations (Extended Data Fig. 2i). These results revealed that whereas T3-LDVax generates tumour-specific CD4^+^ T cells whose products promote help to antitumour responses, T3-HDVax induces tumour-specific CD4^+^ T cells that dampen antitumour activity.

## Identification of inhibitory CD4^+^ TILs by scRNA-seq

**Fig. 3:**
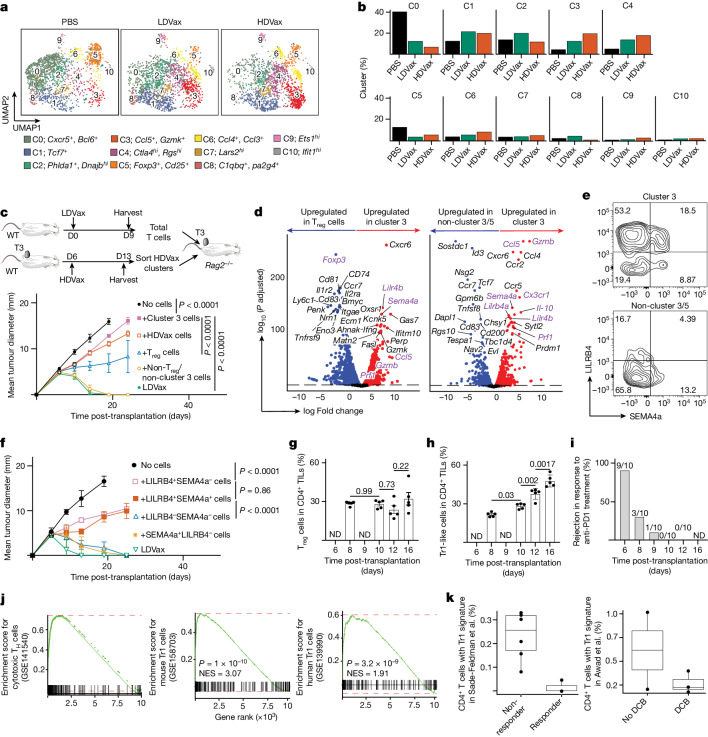
HDVax-induced cells are cytotoxic Tr1 cells that are necessary and sufficient for HDVax-mediated inhibition. **a**, UMAP shows individual clusters in I-A^b^–mItgb1-specific CD4^+^ TILs under different conditions. **b**, Frequency of cells populating individual clusters. **c**, T3 tumour outgrowth in *Rag2*^−/−^ mice that received T cells from LDVax mice plus total CD25^−^CD4^+^ T cells (approximately 0.5 × 10^6^) or different subpopulations (approximately 0.2 × 10^6^) sorted from HDVax-treated mice (*n* = 5). **d**, Volcano plot of bulk RNA-seq comparing gene expression in cluster 3 cells relative to T_reg_ cells (left) and non-cluster 3/5 cells (right). *P* values were obtained after applying the DESeq2 tool. **e**, Representative flow cytometry plot showing LILRB4 and SEMA4a expression in HDVax-induced mItgb1-specific CD4^+^ TILs in T3 tumours. **f**, T3 tumour outgrowth in *Rag2*^−/−^ mice receiving T cells from LDVax mice and different subpopulations (approximately 0.1 × 10^6^) sorted from HDVax-treated mice based on LILRB4 and SEMA4a expression (*n* = 3 (PBS and LDVax) and *n* = 8 for the other groups). **g**,**h**, Frequency of T_reg_ cells (**g**) or LILRB4-expressing CD4^+^ TILs (**h**) in T3 tumours harvested at multiple days post-tumour inoculation (*n* = 5). ND, not done. **i**, Percent of T3 tumour-bearing mice surviving after three doses of anti-PD1 started on different days (6, 8, 9, 10 or 12) post-tumour transplant, followed by two subsequent doses injected every 3 days. The numbers above each bar represent the number of mice that rejected the tumours over the total mice used pooled from two experiments. Data in panels **c**,**f** are expressed as mean tumour diameter ± s.e.m. and are representative of three and two experiments, respectively. Statistics were done using two-way ANOVA, with multiple comparisons corrected using Sidak’s multiple comparison test. Data in panels **g**,**h** are expressed as mean ± s.e.m. representing two experiments; statistics were performed using one-way ANOVA, with multiple comparisons corrected with Tukey’s method. **j**, GSEA comparing the gene signature of cluster 3 cells (bulk RNA-seq) to the gene signature of different CD4^+^ T cell subsets. The red line shows the maximal deviation from zero of the curve, which is basically equal to the enrichment score in these cases. The enrichment score was used to identify the *P* value identification for each gene set by permutation test. NES, normalized enrichment score. **k**, Box plot showing the frequency of Tr1-like cells between responders and non-responders within the Sade-Feldman et al. dataset (left) and durable clinical benefits (DCBs) or non-DCBs within the Awad et al. dataset (right). Statistics were performed using *t*-tests without assuming equal variances. The *P* values in the Sade-Feldman et al. dataset was *P* = 0.002, *t* = 4.98, d.f. = 6.11; no significant difference in the Awad et al. dataset was found (*P* = 0.55). Data are shown as box plots extending from the 25th to 75th percentiles, with the median as the centre and the whiskers corresponding to the minimum and maximum values. Source Data

To better define the HDVax-induced inhibitory CD4^+^ T cells, we performed single-cell RNA sequencing (scRNA-seq) on mItgb1-specific CD4^+^ TILs from mice treated with T3-HDVax, T3-LDVax or PBS. Unbiased uniform manifold approximation and projection (UMAP) clustering of mItgb1-specific CD4^+^ TILs revealed 11 cellular clusters (cluster 0 to cluster C10) with distinct gene signatures (Fig. 3a,b). We found that vaccination per se resulted in a threefold reduction in the frequencies of CD4^+^ TILs populating cluster 0 (follicular helper T (T_FH_) cell cluster (*Bcl6*^+^ and *Cxcr5*^+^)) and cluster 5 (T_reg_ cell cluster (*Foxp3*^+^ and *Cd25*^+^)) in tumours from mice receiving either T3-HDVax or T3-LDVax compared with PBS. By contrast, the proportions of cells populating clusters 1–4, 6 and 8 were differentially affected by the MHC-II peptide dose used in the vaccine. Clusters 2 and 8 were more densely populated following T3-LDVax than following T3-HDVax or PBS treatment, whereas clusters 3, 4 and 6 were more densely populated following T3-HDVax treatment than following LDVax or PBS treatment. Whereas T3-HDVax induced only minor density increases in clusters 4 and 6 compared with T3-LDVax or PBS treatment, the cell density of cluster 3 (*Gzmb*^*+*^*/Ccl5*^*+*^) increased to the greatest extent and showed the largest differences between HDVax versus LDVax or PBS.

**Extended Data Fig. 3:**
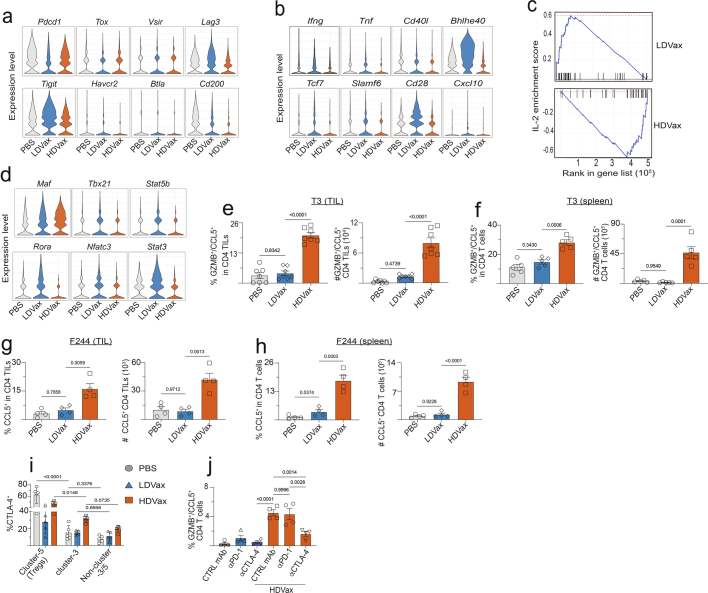
HDVax-induced CD4^+^ T cells are deficient in Th1 gene signature and express high levels of GZMB and CCL5. **a-b**, violin plots showing the expression levels of multiple genes involved in **a**, exhaustion or **b**, effector Th1 signature in mItgb1-specific scRNAseq data. **c**, GSEA showing relative expression of genes involved in the IL-2 signaling pathway. **d**, violing plots showing the expression levels of different transcription factors involved in IL-2 signaling in mItgb1-specific scRNAseq data. **e-f**, frequency **(left)** and numbers **(right)** of CCL5/GZMB expressing CD4^+^ T cells in **e**, TILs and **f**, spleens of T3 tumour bearing WT mice 5-7 days post vaccinations (n = 7 (TILs) and 5 (spleen) and represent three experiments). **g-h**, frequency **(left)** and numbers **(right)** of CCL5/GZMB expressing CD4^+^ T cells in **g**, TILs and **h**, spleens of F244 tumour-bearing WT mice seven days post vaccinations (n = 4, represent two experiments). **i**, frequency of CTLA-4 in different clusters of mItgb1-specific CD4^+^ TILs of T3 tumour-bearing WT mice (n = 5, represent theee experiments). **j**, frequency of CCL5/GZMB expressing CD4^+^ TILs of T3 tumour-bearing WT mice treated with HDVax plus αPD-1 or αCTLA-4 (n = 4, represent three experiments). Data in all panels are shown as means ± s.e.m. Statistics were performed using one-way ANOVA with multiple comparisons corrected with Tukey’s method in **e-h** and **j**, and two-way ANOVA corrected with sídák’s multiple comparisons test was performed for statistics in **i**. Source Data

Additional analyses revealed that T3-HDVax-induced CD4^+^ TILs expressed relatively high levels of *Pdcd1*, *Tox* and *Vsir* transcripts and relatively low levels of *Lag3*, *Cd200, Havcr2* and *Tigit* transcripts compared with those induced by T3-LDVax or PBS (Extended Data Fig. 3a). CD4^+^ TILs from T3-HDVax-treated mice also expressed lower mRNA levels for *Ifng*, *Tnf*, *Cd28* and *Cd40l* and transcription factors associated with T helper 1 (T_H_1) cell polarization, for example, *Bhlhe40*, than TILs from T3-LDVax-treated or PBS-treated mice (Extended Data Fig. 3b). As shown by *Il2* gene set enrichment analysis (GSEA), HDVa-induced cells exhibited defects in the IL-2 signature **(**Extended Data Fig. 3c), expressed low levels of transcription factors such as *Stat5b*, *Rora* and *Nfatc*, and showed elevated expression of the *cMaf* gene that suppresses IL-2 production^22^ compared with corresponding cells following T3-LDVax treatment **(**Extended Data Fig. 3d).

To validate these observations, we assessed the levels of the corresponding proteins in tumour-specific CD4^+^ TILs and splenocyte populations from mice treated with T3-HDVax, T3-LDVax or PBS. These analyses confirmed that GZMB^+^/CCL5^+^ mItgb1-specific CD4^+^ TILs were present at fivefold higher frequencies and numbers in T3-HDVax-treated mice than in TILs from mice treated with T3-LDVax or PBS (Extended Data Fig. 3e,f). A 2–3-fold difference was also noted in splenocytes from mice receiving T3-HDVax compared with T3-LDVax or PBS. Similar results were observed using F244 tumour-bearing mice treated with F244-HDVax, F244-LDVax or PBS (Extended Data Fig. 3g,h). In both T3 and F244 tumour models, the respective HDVax-induced cluster 3 cells expressed higher levels of CTLA4 protein than cells in all other clusters, except cluster 5 (classical CD25^+^ T_reg_ cells; Extended Data Fig. 3i). Treatment with anti-CTLA4 but not anti-PD1 eliminated cluster 3 and cluster 5 cells in TILs of HDVax-treated mice **(**Extended Data Fig. 3j). This finding explains why HDVax did not inhibit antitumour efficacy of anti-CTLA4, because the anti-CTLA4 monoclonal antibody (9D9) used in the study depletes both T_reg_ cells and the HDVax-induced inhibitory CD4^+^ T cells (Fig. 1e,f).

## Cluster 3 cell ACT inhibits tumour rejection

**Extended Data Fig. 4:**
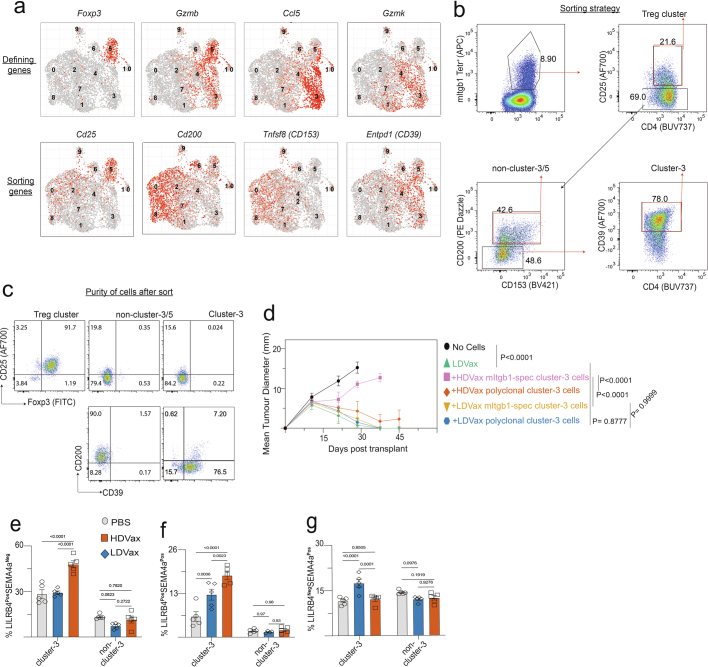
Gating and sorting strategy to patially enrich for cluster-3 cells. **a**, feature plot showing the expression of various genes superimposed on I-A^b^-mItgb1 specific CD4^+^ TILs UMAP**. b**, representative flow cytometry plots showing the sorting strategy for different clusters in mItgb1-specific CD4^+^ TILs. **c**, representative flow cytometry plot showing the expression of CD25, CD39 and Foxp3 in different clusters after sorting. **d**, T3 outgrowth in RAG2^−/−^ mice receiving total T cells from LDVax-treated WT mice (5 × 10^6^) alone or plus; LDVax-induced cluster-3 cells; HDVax-induced, CD25-negative mItgb1-specific; or polyclonal (mItgb1 non-specific) CD4^+^ T cells (~0.2 × 10^6^). Data expressed as mean tumour diameter ± s.e.m. (n = 5, two experiments). *P* values were calculated using Two-way ANOVA corrected with sídák’s multiple comparisons test. **e-g**, frequency of LILRB4 single positive, LILRB4^/^SEMA4a double positive or SEMA4a single positive expressing mItgb1-specific CD4^+^ TILs (n = 5, represent three experiments). Data are expressed as mean ± s.e.m. Statistics were performed using one-way ANOVA with multiple comparisons corrected with Tukey’s method. Source Data

Although these results suggested that the inhibitory HDVax-induced cells of interest were localized to cluster 3, they did not identify surface proteins on these cells that would permit their isolation. We therefore established a sorting strategy in which we removed T cells from the tumour-specific CD4^+^ T cell population that expressed genes of surface markers not found in cluster 3 cells (that is, *Cd25*, *Cd200* and *Cd153*), and positively sorted the remaining cells for CD39 expression (Extended Data Fig. 4a). This approach allowed us to generate three cellular pools: (1) partially purified cluster 3 cells (CD25^−^CD200^−^CD153^−^ and CD39^+^), (2) CD25^+^ cluster 5 T_reg_ cells (more than 90% FOXP3^+^), and (3) non-cluster 3/5 cells (CD25^−^CD200^+^CD153^+^ and CD39^−^; Extended Data Fig. 4b,c). These cellular pools were then tested for their capacity to inhibit T3 rejection. T3 tumours were rejected in *Rag2*^−/−^ mice receiving T_reg_ cell-depleted T3-LDVax-induced effector T cells either alone or in combination with HDVax-induced, T3-specific non-cluster 3/5 cells (Fig. 3c). By contrast, mixtures of LDVax-induced effector T cells plus HDVax-induced T3-specific cluster 3 cells did not reject T3 tumours (Fig. 3c). Similar mixtures containing classical cluster 5 T_reg_ cells instead of cluster 3 cells only partially inhibited tumour rejection. As expected, mixtures of T3-LDVax-induced effector T cells plus either LDVax-induced T3-specific cluster 3 cells or polyclonal CD4^+^ T cells from T3-HDVax-treated mice lacking mItgb1 specificity did not suppress T3 rejection (Extended Data Fig. 4d). Thus, tumour-specific HDVax-induced cells populating cluster 3 are both necessary and sufficient to mediate inhibition of antitumour efficacy.

## LILRB4 is a marker of inhibitory CD4^+^ T cells

To better define the immunosuppressive cells populating cluster 3 and derive insights into their origins and mechanism of action, we subjected isolated HDVax-induced cluster 3, cluster 5 (T_reg_ cells) and non-cluster 3/5 cell populations to deep RNA-seq and compared their transcriptomes. Volcano plot analyses confirmed that cluster 3 cells expressed high levels of *Gzmb* and *Ccl5* (Fig. 3d). This characteristic was not shared either by T_reg_ cells or non-cluster 3/5 cells. T3-HDVax-induced cluster 3 cells also showed selective enrichment in the expression of two surface markers known to have immunosuppressive activities: *Lilrb4* (encoding ILT3, CD85k or gp49b)^23–26^ and *Sema4a*^27,28^. Flow cytometry analyses revealed that HDVax cluster 3 cells could be divided into four subpopulations based on single or dual expression of LILRB4 and SEMA4a proteins (Fig. 3e and Extended Data Fig. 4e–g). Adoptive transfer of HDVax-induced LILRB4^+^SEMA4a^−^ or LILRB4^+^SEMA4a^+^ mItgb1-specific CD4^+^ T cells comparably ablated T3 tumour rejection in the *Rag2*^−/−^ ACT assay (Fig. 3f). By contrast, adoptively transferred SEMA4a single-positive cells or double-negative cells did not inhibit tumour rejection.

As these results established surface markers that identified the inhibitory cells, we asked whether they were present in progressively growing tumours that became insensitive to anti-PD1. Indeed, the frequency of FOXP3^−^LILRB4^+^ TILs increased in progressively growing, untreated T3 tumours (Fig. 3g,h), and their appearance correlated with the onset of resistance to anti-PD1 (Fig. 3i). Similar increases in T_reg_ cells were not observed under these conditions. These results, together with the scRNA-seq data, reveal that LILRB4 expression identifies a non-T_reg_ cell suppressive CD4^+^ T cell population induced by HDVax that also develops in untreated progressively growing tumours as they develop ICT insensitivity.

## HDVax-induced cells are Tr1 cells

**Extended Data Fig. 5:**
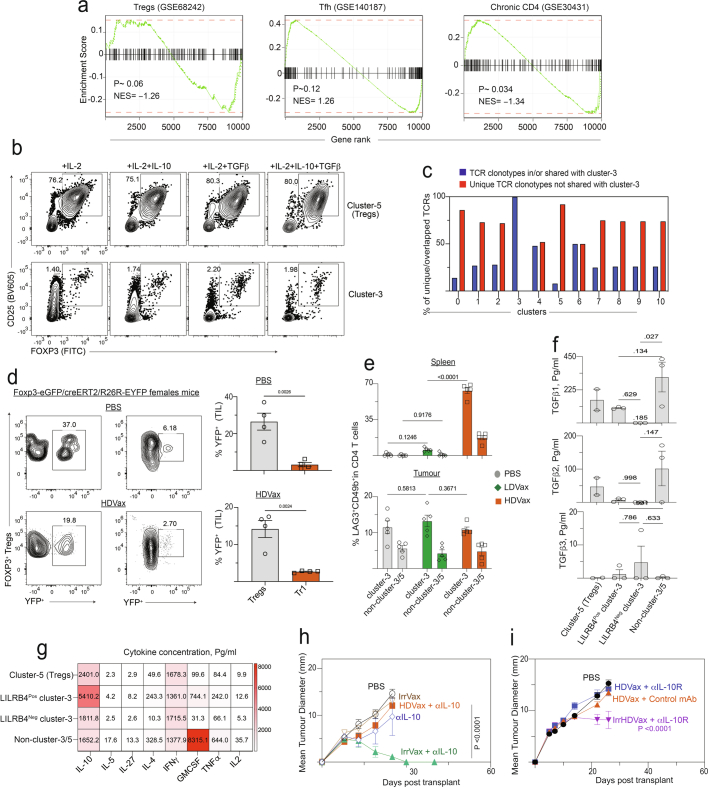
Cells populating cluster-3 are distinct from Tregs. **a**, GSEA comparing gene signature of cluster-3 cells (bulk-RNAseq) to gene signature of different CD4^+^ T cell subsets. Enrichment Score was used to identify the p-value identification for each gene set by permutation test. **b**, flow cytometry plot showing CD25 and Foxp3 staining in sorted Tregs and cells populating cluser-3 after 10 days of in vitro incubation with IL-2 alone (30 IU/ml), or plus IL-10 and or TGFβ (10 ng/ml). The experiment represents two experiments. **c**, TCR clonotypes in each cluster of HDVax-induced I-A^b^-mItgb1 specific CD4^+^ T were determined by TCRseq and compared to the TCR clonotypes in cells populating cluster-3. **d**, frequency of cells expressing YFP^+^ in Tregs (CD25^+^Foxp3^+^) or CD25^−^LILRB4^+^ CD4^+^ T cells after different treatments in spleens and TILs (n = 4, represent one experiment). **e**, frequency of cells expressing LAG3 and CD49b in cluster-3 or non-cluster-3/5 of mItgb1-specific CD4^+^ T cells after different treatments in spleens and TILs (n = 5, represent three experimetns). Data expressed as mean tumour diameter ± s.e.m. and statistics were performed by two-way ANOVA corrected with sídák’s multiple comparisons test. **f-g**, freshly isolated CD4^+^ T cells from different clusters were stimulated with 1 μg/ml mItgb1 SLP for 72 hr, and production of active isoforms of TGFβ was assessed by Multiplex (biological replicates = 2 in cluster-5 and 3 replicates in all other clusters and each values are mean of 2 technical replicates, represent two experiments). Statistics in **d** and **f** were performed using one-way ANOVA with multiple comparisons corrected with Tukey’s method. **g**, Heatmap showing levels of different cytokines production (indicated on the heatmap x-axis). Numbers represent the mean of three independent biological replicates and represent two experiments. **h**, T3 tumour outgrowth in WT mice treated with HDVax or irrelevant vaccines on day 6 and αIL-10 (-1, 4, 7, 10 and 14) post-T3 tumour injection (n = 5, representing two experiments). **i**, T3 tumour outgrowth in WT mice treated with HDVax on day 3 and αIL-10R (-1, 5, 11 and 18) post tumour transplantation (n = 10, representing one experiment). Data in **e**, **h** and **i** expressed as mean tumour diameter ± s.e.m. and *P* values were calculated using two-way ANOVA corrected with sídák’s multiple comparisons test. Source Data

GSEA revealed that cluster 3 cells display a distinctive gene signature differentiating them from FOXP3^+^ T_reg_ cells^29^, T_FH_ cells^30^ and chronically stimulated exhausted CD4^+^ T cells^31^ (Extended Data Fig. 5a). HDVax-induced inhibitory cells remained FOXP3^−^ after 10 days of incubation with different cytokines in vitro (Extended Data Fig. 5b). In addition, HDVax-induced cells were not derived from T_reg_ cells as evidenced by lack of shared TCR clonotypes populating cluster 3 and cluster 5 and in vivo lineage-tracing studies using FOXP3 lineage-tracing mice^32^ (Extended Data Fig. 5c,d). Thus, cluster 3 cells were neither precursors of, nor differentiated from, T_reg_ cells. Conversely, the gene signature of cluster 3 cells significantly overlapped those reported for mouse and human Tr1 cells^33–35^, and cytotoxic CD4^+^ T cells^36^ (Fig. 3j). In addition, the proportion of Tr1-like cells was higher in patients displaying resistance to anti-PD1 therapy^37^ and personalized cancer vaccines^38^ (Fig. 3k). Whereas expression of LAG3 and CD49b (sometimes used to define Tr1 cells^39^) were not different in TILs of HDVax-treated versus LDVax-treated mice, these markers were selectively expressed on mItgb1-specific CD4^+^ T cells in the spleens of T3-HDVax-treated mice, but not in T3-LDVax-treated mice (Extended Data Fig. 5e). T3-HDVax-induced LILRB4^+^ cluster 3 cells produced high amounts of IL-10 and low amounts of IL-2 (hallmarks of Tr1 cells), as well as low levels of TNF and GM-CSF compared with LILRB4^−^ cluster 3 cells, T_reg_ cells or non-cluster 3/5 cells (Extended Data Fig. 5g). TGFβ was produced mainly by non-cluster 3/5 cells and T_reg_ cells (Extended Data Fig. 5f). IL-10 blockade using anti-IL-10 or anti-IL-10R did not rescue HDVax inhibition (Extended Data Fig. 5h,i), indicating that IL-10 was not sufficient to impede antitumour efficacy. These data suggest that HDVax and progressively growing tumours that become insensitive to anti-PD1 therapy do so partly because of elaboration of inhibitory FOXP3^−^CD4^+^ Tr1 cells.

## Anti-LILRB4 reverses suppression by Tr1-like cells

**Fig. 4:**
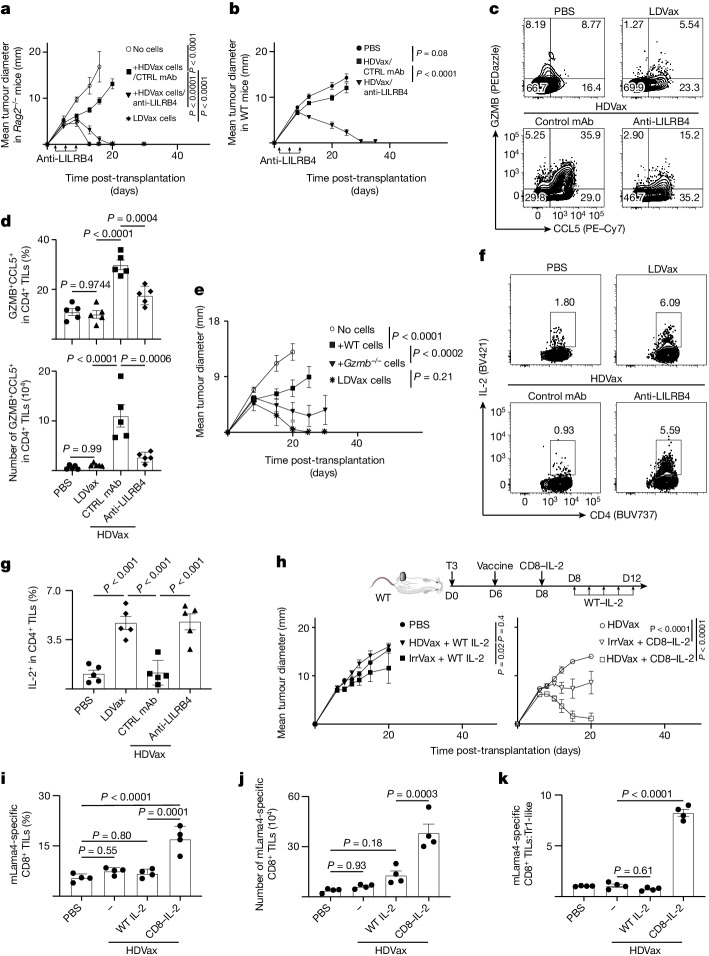
Anti-LILRB4 treatment reverses HDVax induction of GZMB/CCL5 and suppression of IL-2 production. **a**, T3 tumour outgrowth in *Rag2*^−/−^ mice receiving T cells from LDVax-treated mice plus HDVax-induced, CD25-negative mItgb1-specific CD4^+^ T cells (approximately 0.5 × 10^6^) followed by anti-LILRB4 or control (CTRL) monoclonal antibody (mAb) treatment (*n* = 3 (PBS), 5 (LDVax and anti-LILB4) and 8 for control mAb). **b**, T3 tumour outgrowth in WT mice treated with HDVax alone or plus anti-LILRB4 (*n* = 5 (PBS) and 10 for other groups). **c**, Representative flow cytometry plots showing GZMB/CCL5 expression in CD4^+^ TILs pregated on CD4^+^CD25^−^FOXP3^−^CD39^+^. **d**, Frequency (top) and numbers (bottom) of GZMB/CCL5-expressing CD4^+^ TILs in T3 tumours 7 days after treatments (*n* = 5). **e**, 1956 Tumour outgrowth in *Rag2*^−/−^ mice receiving T cells from LDVax WT mice and HDVax-induced, CD25-negative mItgb1-specific CD4^+^ T cells (approximately 0.2 × 10^6^) from WT or *Gzmb*^−/−^ mice (*n* = 5 for PBS and LDVax, and 7 for other groups). **f**, Representative flow cytometry plots show the IL-2 expression in CD4^+^ TILs. **g**, Frequency of cells expressing IL-2 in CD4^+^ T cells in multiple mice (*n* = 5). **h**, T3 tumour outgrowth in WT mice treated as shown and detailed in the Methods (*n* = 5). **i**–**k**, T3 tumour-bearing mice were treated as in panel **h**, and the frequency (**i**) and numbers (**j**) of mLama4-specific CD8^+^ TILs, and the ratio of mLama4-specific CD8^+^ TILs to Tr1-like cells (**k**) were assessed on day 13 TILs (*n* = 4). The data in panels **a**,**b**,**e**,**h** are expressed as mean tumour diameter ± s.e.m. and represent three, two, two and three experiments, respectively; statistics were done using two-way ANOVA, with multiple comparisons corrected using Sidak’s multiple comparison test. Data in panels **d**,**g**,**i**–**k** are shown as mean ± s.e.m. representing three, three, two and three experiments, respectively; statistics were done using one-way ANOVA, with multiple comparisons corrected with Tukey’s method. Source Data

**Extended Data Fig. 6:**
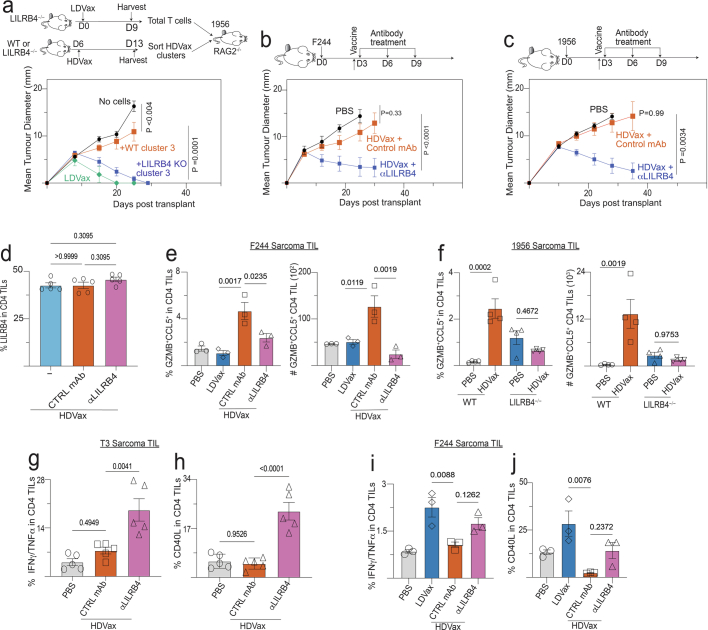
related to Fig. 4: αLILRB4 treatment reverses HDVax induction of GZMB/CCL5 and suppression of IL-2 production. **a**, 1956 outgrowth in RAG2^−/−^ mice receiving total LILRB4^−/−^ T cells from LDVax-treated LILRB4^−/−^ mice plus HDVax-induced, CD25-negative CD4^+^ T cells (~0.2 × 10^6^) from WT mice or LILRB4^−/−^ mice (n = (5 for PBS and LDVax) 7 for other groups). **b**, F244 tumour outgrowth in WT mice treated with HDVax and αLILRB4 as shown (n = 10, represent two experiments). **c**, 1956 tumour outgrowth in mice treated with HDVax and αLILRB4 as shown (n = 10, represent two experiments). **d**, frequency of cells expressing LILRB4 in CD4^+^ T3 TILs after treatment with αLILRB4 or control mAb (n = 5, represent three experimenrts). **e**, frequency and numbers of GZMB/CCL5 expressing CD4^+^ TILs in F244 tumour-bearing mice (n = 3, represent two experiments). **f**, frequency and numbers of GZMB/CCL5 expressing CD4^+^ TILs in WT and LILRB4^−/−^ 1956 tumour-bearing mice (n = 4, represent two experiments). **g-h**, frequency of CD4^+^ TILs expressing **g**, IFN-γ/TNF-α or **h**, CD40L in T3 TILs after in vitro activation with mItgb1 peptide in the presence of Golgi-plug (n = 5, represent two experiments). **i-j**, frequency of CD4^+^ TILs expressing **i**, IFN-γ/TNF-α or **j**, CD40L in F244 TILs after in vitro activation with mPlec peptide in the presence of Golgi-plug (n = 3). Data in **a-c** expressed as mean tumour diameter ± s.e.m and *P* values were calculated using Two-way ANOVA corrected with sídák’s multiple comparisons test. Data in **d-j** are expressed as mean ± s.e.m. and *P* values were calculated using one-way ANOVA with multiple comparisons corrected with Tukey’s method. Source Data

On the basis of the finding that inhibitory Tr1 cells expressed LILRB4 protein on their surface, we asked whether a recently reported blocking LILRB4 monoclonal antibody^23^ could abrogate the suppressive actions of these cells. As expected, in *Rag2*^−/−^ mice, transfer of T3-LDVax-induced effector T cells induced T3 rejection, and mixtures of effector T cells plus T3-HDVax-induced Tr1 cells inhibited rejection (Fig. 4a). Whereas addition of control monoclonal antibody to the latter mixture did not reverse inhibition of tumour rejection, addition of anti-LILRB4 facilitated tumour rejection. Similarly, whereas transfer of HDVax-induced cluster 3 cells from WT mice suppressed tumour rejection in this assay, transfer of CD25^−^CD200^−^CD39^+^ cluster 3 cells from *Lilrb4*^−/−^ mice did not inhibit tumour rejection (Extended Data Fig. 6a). Moreover, T3 tumours grew progressively in WT mice treated with T3-HDVax and control monoclonal antibody, but were rejected in WT mice receiving the combination of T3-HDVax + anti-LILRB4 (Fig. 4b). Similar results were observed using the F244 and 1956 sarcoma models with their respective HDVax treatments (Extended Data Fig. 6b,c, respectively).

We next asked whether anti-LILRB4 treatment provoked phenotypic and/or functional changes in tumour-specific HDVax-induced Tr1 cells. We confirmed that anti-LILRB4 did not deplete LILRB4-expressing CD4^+^ TILs in vivo^23^ (Extended Data Fig. 6d). Treatment with this monoclonal antibody decreased levels of GZMB/CCL5 expressed per CD4^+^ T cell harvested from T3 (Fig. 4c,d) or F244 tumour**-**bearing mice (Extended Data Fig. 6e). A similar reduction of GZMB/CCL5-expressing CD4^+^ TILs was observed when a comparison was made between 1956 tumour-bearing WT mice and mice with a targeted disruption of the *Lilrb4* gene (Extended Data Fig. 6f). These results implied a connection between cellular expression of LILRB4 and GZMB.

To test the role of GZMB in the Tr1-dependent inhibitory process, we performed the *Rag2*^−/−^ ACT assay using cluster 3 cells derived from T3-HDVax-treated WT versus *Gzmb*^−/−^ mice after first confirming similar levels of LILRB4 expression in cells derived from each strain. Whereas T3-HDVax-induced cluster 3 cells from WT mice displayed potent inhibition of tumour rejection, T3-HDVax-induced *Gzmb*^−/−^ cluster 3 cells displayed reduced inhibitory capacity (Fig. 4e).

**Extended Data Fig. 7:**
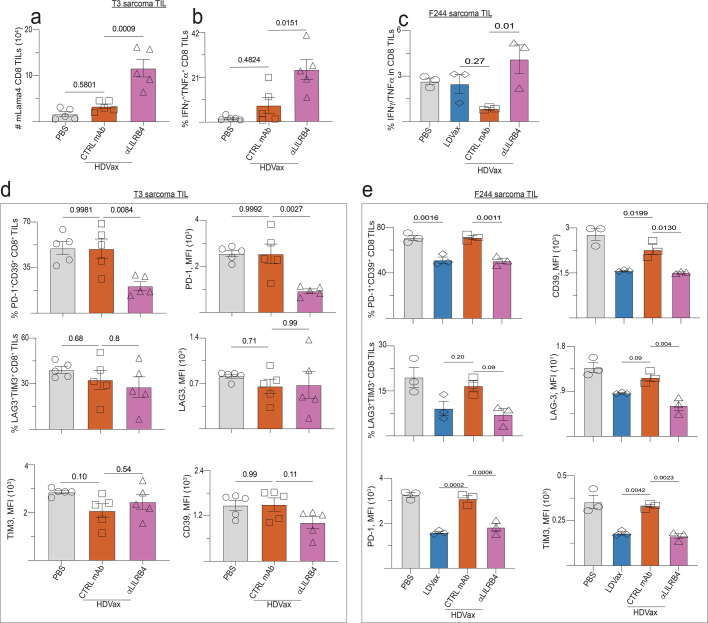
LILRB4 blockade enhances the phenotype and the effector functions of CD8^+^ TILs. **a**, numbers of mLama4^+^ specific CD8^+^ TILs were assessed in T3 TILs (n = 5). **b**, frequency of CD8^+^ TILs expressing IFN-γ/TNF-α after in vitro activation with mLama4 SLP. **c**, frequency of CD8^+^ TILs expressing IFN-γ/TNF-α after in vitro activation with mPex14 SLP (n = 3). **d**, expression of several exhaustion markers on mLama4-specific CD8^+^ T cells in TILs of T3 tumour-bearing mice (n = 5). Data represent two independent experiments and are shown as mean ± s.e.m. **e**, expression of several exhaustion markers in mPex14-specific CD8^+^ T cells in F244 TILs (n = 3). Data expressed are shown as mean ± s.e.m. *P* values were calculated using one-way ANOVA with multiple comparisons corrected with Tukey’s method, which was used for the statistics. Source Data

We then assessed the capacity of anti-LILRB4 to affect the induction of other CD4^+^ T cell-derived products that provide help to antitumour responses. LILRB4 blockade restored IL-2 production in HDVax-induced cells (Fig. 4f,g) and enhanced their expression of IFNγ/TNF and CD40L in both T3 and F244 tumour models (Extended Data Fig. 6g,j). Concomitant with these changes, anti-LILRB4 increased the number of mLama4-specific CD8^+^ TILs and decreased expression of the exhaustion markers PD1, LAG3 and TIM3. Similar findings were recapitulated using F244 tumours and F244-HDVax (Extended Data Fig. 7a–e). Thus, in mice, the inhibitory Tr1-like cells are marked by expression of LILRB4, and their inhibitory capacity is reversed either by monoclonal antibody blockade or genetic deletion of the protein that, in turn, reduces the expression of GZMB and increases expression of IL-2 and other molecules that facilitate antitumour efficacy.

## CD8–IL-2 circumvents HDVax suppression

**Extended Data Fig. 8:**
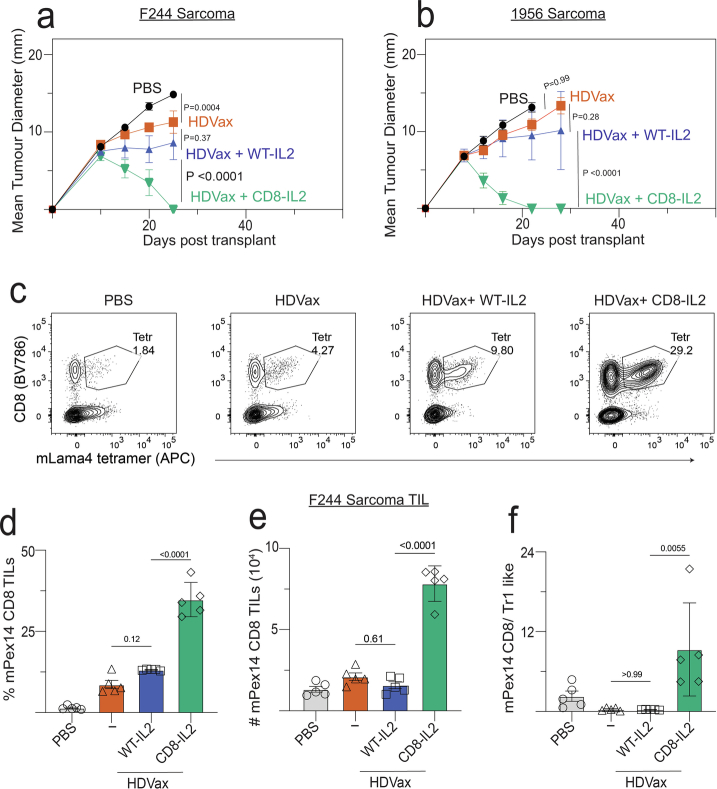
related to Fig. 4: CD8-directed, cis-targeted IL-2 treatment re-establishes the anti-tumour efficacy of HDVax in F244 and 1956 tumour models. **a**, F244 tumour outgrowth in WT mice treated as in Fig. 4h (n = 10, represent two experiments). **b**, 1956 tumour growth in C57BL6 WT mice treated as in Fig. 5i (n = 5, represent two experiments). Data in **a** and **b** are expressed as mean ± s.e.m., and *P* values were calculated using Two-way ANOVA corrected with sídák’s multiple comparisons test. **c**, representative flow cytometry plot showing the expression of mLama4^+^ specific CD8^+^ TILs in T3 TILs harvested from treated mice, as shown in Fig. 5h. **d-f**, F244 tumour-bearing mice were treated as in Fig. 5h, and **d**, frequency, **e**, numbers of mPex14-specific CD8^+^ TILs, and **f**, the ratio of mPex14-specific CD8^+^ TILs to Tr1-like cells were assessed on day 13 TILs. Data are expressed as mean ± s.e.m., and *P* values were calculated using one-way ANOVA with multiple comparisons corrected with Tukey’s method. Source Data

The ability of anti-LILRB4 to restore IL-2 production in HDVax-induced CD4^+^ T cells prompted us to ask whether IL-2 supplementation would restore effector CD8^+^ T cell function and tumour rejection. Although WT IL-2 is an approved therapy for certain cancers, its clinical use is limited due to its pleiotropic effects and high toxicity^40^. We therefore utilized a CD8^+^ T cell *cis*-targeted IL-2 mutein (CD8–IL-2) that binds selectively to CD8^+^ T cells but not to T_reg_ cells, natural killer cells or conventional CD4^+^ T cells^41^. CD8–IL-2 selectively expands CD8^+^ T cells in mice and primates and maintains CTL function without inducing toxicity^41^. Neither T3-HDVax nor subtherapeutic doses of CD8–IL-2 (0.3 mg kg^−1^) induced tumour rejection in T3-bearing mice as monotherapies. By contrast, the combination of T3-HDVax plus subtherapeutic doses of CD8–IL-2 drove T3 tumour rejection (Fig. 4h). Combining IrrVax with CD8–IL-2 was ineffective. Treatment with the combination of WT IL-2 and either T3-HDVax or Irr-HDVax did not induce tumour rejection but resulted in significant toxicity, evidenced by weight loss in treated mice. Similar findings were observed using the F244 and 1956 tumour models (Extended Data Fig. 8a,b, respectively).

Following treatment with HDVax plus CD8–IL-2, a 2.5-fold increase in frequency (Fig. 4i and Extended Data Fig. 8c) and absolute numbers (Fig. 4j) of mLama4-specific CD8^+^ TILs was observed. In addition, CD8–IL-2 treatment was associated with significant increases in the ratio of CD8^+^ TILs to Tr1-like cells (Fig. 4k). Similar findings were also observed in the F244 tumour model (Extended Data Fig. 8d–f). Thus, targeting an IL-2 mutein to CD8^+^ T cells circumvents HDVax inhibition by increasing the frequency of antigen-specific cytotoxic CD8^+^ T cells, leading to tumour regression.

## Induction of inhibitory Tr1 cells requires cDC2s/monocytes

**Fig. 5:**
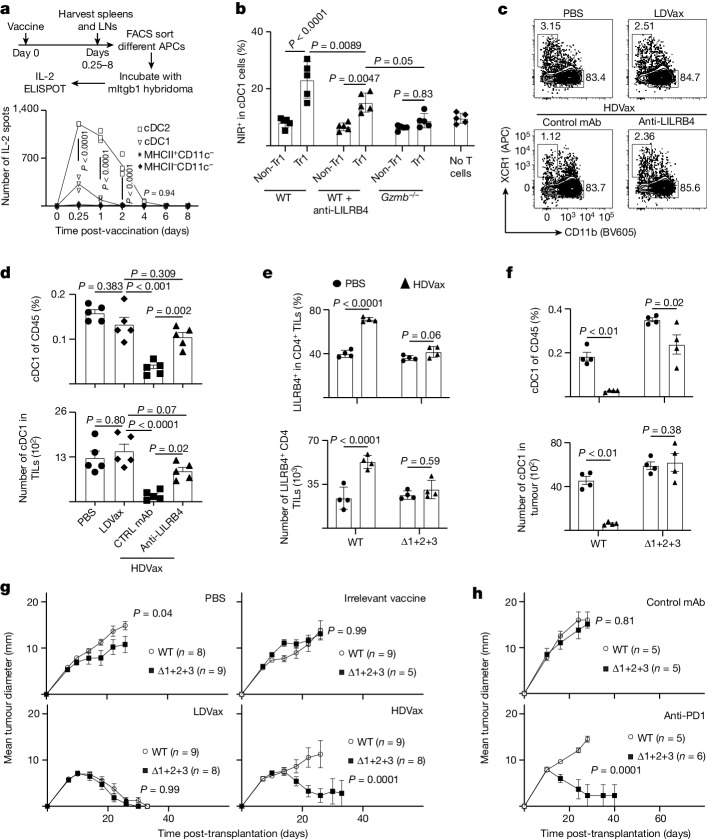
HDVax-induced Tr1-like cells arise from preferential cDC2 antigen presentation and kill cDC1 in a GZMB-dependent manner. **a**, Sorting protocol and IL-2 production by mItgb1-specific hybridoma (*n* = 3, representing two experiments). **b**, Frequency of NIR expression in cDC1 (MHCII^+^CD11c^+^XCR1^+^) after incubation with sorted subpopulations of HDVax-induced CD4^+^ T cells harvested from WT mice (treated with HDVax plus control mAb or anti-LILRB4) or *Gzmb*^−/−^ mice (*n* = 5, representing three experiments). **c**, Representative flow cytometry plots showing cDC1 and cDC2 in T3 tumours pregated on CD45^+^MHC-II^+^CD11c^+^ and CD64^−^. **d**, Frequency (top) and numbers (bottom) of cDC1 in T3 tumours assessed 5 days post-vaccination (*n* = 5, representing three experiments). **e**, Frequency (top) and numbers (bottom) of LILRB4^+^CD25^−^FOXP3^−^CD4^+^ T cells in 1956 TILs harvested from WT or Δ1+2+3 tumour-bearing mice after vaccination (*n* = 4, representing three experiments). **f**, Frequency (top) and numbers (bottom) of cDC1 in 1956 tumours in WT and Δ1+2+3 mice assessed 5 days post-vaccination (*n* = 4, representing three experiments). **g**, 1956 Tumour outgrowth in WT and Δ1+2+3 mice vaccinated with 1956 HDVax, LDVax or IrrVax on days 5 and 16. **h**, 1956 Tumour outgrowth in WT and Δ1+2+3 mice treated with 200 μg of anti-PD1 on days 12, 15 and 18 (*n* = 5–6, representing two experiments). Data in panels **a**,**b**,**d**–**f** are expressed as mean ± s.e.m. Data in panels **g**,**h** are expressed as mean tumour diameter ± s.e.m. Statistics in panel **d** were done using one-way ANOVA, with multiple comparisons corrected with Tukey’s method. Statistics in panels **a**,**b**,**e**–**h** were determined by two-way ANOVA, with multiple comparisons corrected using Sidak’s multiple comparison test. Source Data

**Extended Data Fig. 9:**
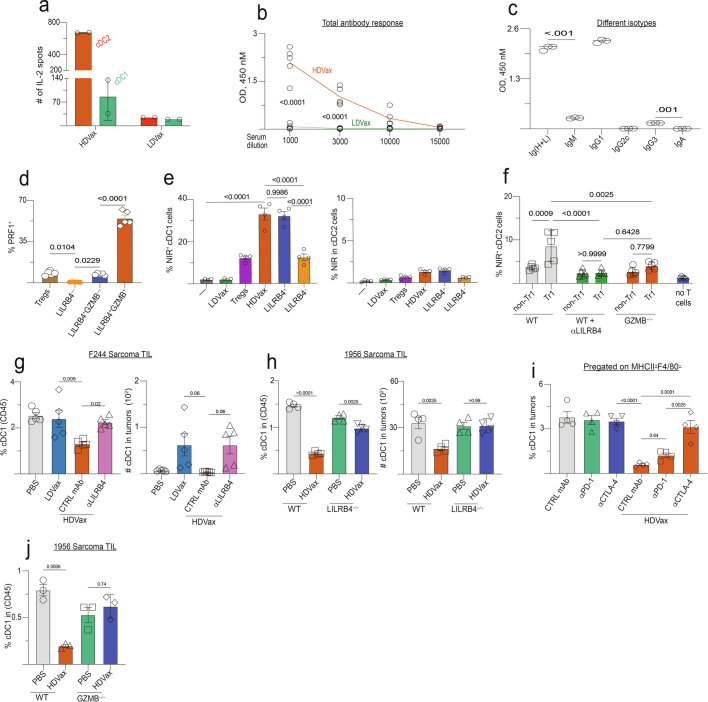
related to Fig. 5: HDVax-induced Tr1-like cells kill cDC1 in a GZMB-dependent manner. **a**, IL-2 production by mItgb1-specific hybridoma cells after incubation with cDC1 and cDC2 sorted from five mice one day after treatment with T3- LDVax or -HDVax (n = 2 and represent two experiments). **b**, T3 tumour-bearing mice were vaccinated with LDVax or HDVax (2 doses,11 days apart), serum was collected 7 days after the last vaccination, and antibody titer to mItgb1 SLP was assessed using ELISA **(Methods)**. **c**, different isotypes of mItgb1-specific antibodies were determined. Data in **b** and **c** are shown as means ± s.e.m. and represent three independent experiments (n = 5). **d**, frequency of perforin (Prf1) expressing CD4^+^ TILs in different subpopulations of CD4^+^ TILs (n = 5, represent two experiments). **e**, frequency of NIR expression (live/dead signal) in cDC1 (MHCII^+^CD11c^+^XCR1^+^) **(left)** and cDC2 (MHCII^+^CD11c^+^CD172a^+^) **(right)** after incubation with sorted LDVax-induced or subpopulations of HDVax-induced mItgb1-specific CD4^+^ T cells **(Methods)**. Data are shown as means ± s.e.m. of three experiments. **f**, frequency of cDC2 (MHCII^+^CD11c^+^CD172a^+^) expressing NIR after incubation with sorted subpopulations of HDVax-induced mItgb1 specific CD4^+^ T cells harvested from WT mice (treated with HDVax plus control mAb or αLILRB4) or GZMB^−/−^ mice (**related to** Fig. 5b). **g**, frequency **(left)** and numbers **(right)** of cDC1 (of CD45^+^ cells) in F244 TILs treated with HDVax five days post vaccinations (n = 5). Data are representative of two independent experiments and shown as mean ± s.e.m. **h**, frequency **(left)** and numbers **(right)** of cDC1 in 1956 TILs assessed five days post-vaccination in WT and LILRB4^−/−^ mice (n = 4). **i**, frequency of cDC1 in T3 tumours assessed five days post-vaccination and treatment with 2 doses of αPD-1 or αCTLA-4 in WT mice (n = 4, represent two experiments). **j**, frequency of cDC1 in 1956 TILs assessed five days post-vaccination in WT and GZMB^−/−^ mice (n = 3). Data in all panels are shown as means ± s.e.m. and *P* values were calculated using one-way ANOVA with multiple comparisons corrected with Tukey’s method. Source Data

We considered the possibility that the different outcomes between HDVax and LDVax might reflect differences in antigen-presenting cells (APCs) that present the mItgb1 neoAg. We explored this possibility by vaccinating naive mice with either T3-LDVax or T3-HDVax, isolating different APC populations at different time points and testing their capacity to present mItgb1 to a mItgb1-specific T cell hybridoma^8^. Using T3-LDVax, we observed that both cDC1s and cDC2s presented the mItgb1 neoepitope to approximately the same extent (Extended Data Fig. 9a). By contrast, using T3-HDVax, the cDC2 population emerged as the primary APCs (Fig. 5a). Consistent with this result, HDVax but not LDVax elicited robust antibody responses to the mItgb1 peptide (Extended Data Fig. 9b,c). As expected, HDVax-induced Tr1 cells expressed high levels of GZMB and perforin (Fig. 3c and Extended Data Fig. 9d), suggesting that they might manifest at least some of their inhibitory function by killing an MHC-II-expressing APC^42^. As T3 cells never express MHC-II, we considered the possibility that the target cells for cytotoxic Tr1 might be cDC1 because they present both MHC-I and MHC-II tumour epitopes^43^ and are, therefore, particularly important to the antitumour response. In vitro killing assays showed that T3-HDVax-induced Tr1 cells but not non-Tr1 CD4^+^ T cells killed mItgb1-pulsed cDC1s but not cDC2s in a GZMB-dependent manner (Fig. 5b and Extended Data Fig. 9e,f). Of note, Tr1 cells harvested from T3-HDVax-treated mice in the presence of anti-LILRB4 showed reduced in vitro killing activity. In addition, the frequency and number of cDC1s recovered from T3-HDVax-treated tumour-bearing mice were reduced by 4.5-fold compared with mice treated with T3-LDVax or PBS. cDC1 recovery was partially restored when T3-HDVax was combined with anti-LILRB4 treatment (Fig. 5c,d and Extended Data Fig. 9g) or when HDVax was administered to *Lilrb4*^−/−^ mice (Extended Data Fig. 9h). Similarly, treatment with anti-CTLA4, which depletes Tr1 cells and T_reg_ cells (Extended Data Fig. 3j), restored recovery of cDC1s in T3-HDVax mice (Extended Data Fig. 9i). No reduction in cDC1 recovery was observed in T3-HDVax-treated *Gzmb*^−/−^ mice (Extended Data Fig. 9j). Thus, vaccines containing high doses of MHC-II neoAg favour antigen presentation predominantly by cDC2s, leading to the formation of cytotoxic Tr1 cells that kill tumour-antigen-presenting cDC1s in a GZMB-dependent manner.

## HDVax protects mice lacking cDC2s/monocytes

**Extended Data Fig. 10:**
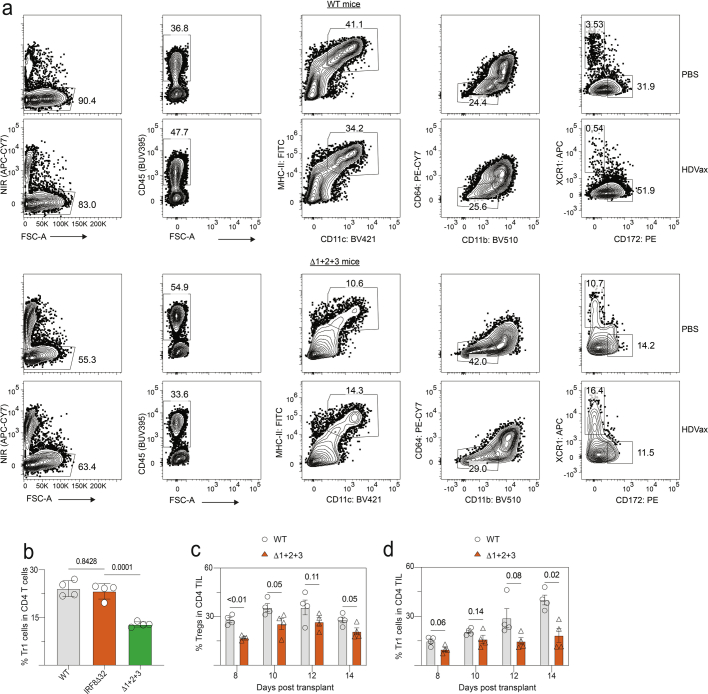
related to Fig. 5: HDVax-induced Tr1-like cells arise from preferential cDC2 antigen presentation. **a**, representative flow cytometry plots showing the gating strategy for different antigen-presenting cell subsets in WT and Δ1 + 2 + 3 mice bearing 1956 tumours. **b**, frequency of Tr1-like cells in spleens of HDVax treated WT, Δ1 + 2 + 3 (cDC2 deficient) or Irf8Δ32 (cDC1 deficient) mice (n = 4, represent two experiments). Data are shown as means ± s.e.m. and *P* values were calculated using one-way ANOVA with multiple comparisons corrected with Tukey’s method. **c**, frequency of Tregs and **d**, Tr1-like cells in 1956 TIL harvested at multiple days post tumour inoculation (n = 4, representing two experiments). Data are expressed as means ± s.e.m. and *P* values were calculated using Two-way ANOVA corrected with sídák’s multiple comparisons test. Source Data

We hypothesized that the genetic elimination of cDC2s/monocytes in mice might reduce the generation of Tr1 cells and ablate the suppressive effect of HDVax. We therefore injected 1956 sarcoma cells into either C57BL/6 WT mice or C57BL/6 mice lacking cDC2s/monocytes (Δ1+2+3) but still capable of generating cDC1s^44,45^. In contrast to WT mice, 1956-HDVax-treated Δ1+2+3 mice showed neither an increase in the frequency of Tr1 cells (Fig. 5e) nor a reduction in cDC1 recovery (Fig. 5f). As expected, 1956 tumours grew progressively in either untreated or Irr-HDVax-treated WT or Δ1+2+3 mice, and were rejected in both strains when tumour-bearing mice were treated with 1956-LDVax. 1956-HDVax induced tumour rejection in Δ1+2+3 mice but not in WT mice (Fig. 5g). Of note, the frequency of Tr1 cells was comparable in both WT mice and in mice lacking cDC1s (IRF8Δ32 mice)^46^ (Extended Data Fig. 10b).

As we observed increased Tr1 cells in progressively growing sarcomas as they lost sensitivity to anti-PD1 (Fig. 3g–i), we compared the appearance of these cells in 1956 tumours growing in WT versus Δ1+2+3 mice. Tr1 cells accumulated in progressively growing 1956 tumours in WT mice as they became insensitive to anti-PD1. By contrast, Tr1 cells did not accumulate in 1956 tumours growing in Δ1+2+3 mice (Extended Data Fig. 10c,d). Finally, anti-PD1 induced the rejection of 12-day-established 1956 tumours in Δ1+2+3 mice but not in WT mice (Fig. 5h), suggesting that the enhanced efficacy of anti-PD1 in the absence of cDC2s and/or monocytes was manifested, at least in part, by the failure to generate cytotoxic Tr1 cells. These results reveal that under HDVax conditions, cDC2s and/or monocytes induce cytotoxic Tr1 CD4^+^ T cells that kill or compromise the integrity/stability of cDC1s, leading to defects in the development/maturation of cytotoxic CD8^+^ T cell responses, thereby compromising antitumour effects of immunity.

## Discussion

The work presented here shows that the effectiveness of therapeutic tumour-specific neoAg peptide vaccines heavily depends on the dose of the MHC-II neoAg contained in the vaccine. We demonstrated that overdosing an MHC-II neoepitope expressed by the tumour in the vaccine can completely reverse vaccine efficacy. This observation is particularly timely and important as therapeutic cancer vaccines are now being actively pursued as personalized cancer immunotherapy^19,47–51^. We do not yet understand the rules governing the amounts of MHC-II neoAgs needed for optimal vaccine effectiveness, nor are we proposing that this needs to be empirically determined for the tumour of each patient. Rather, we focus on defining the nature of the inhibitory CD4^+^ T cell generated under ineffective vaccination conditions and then validate strategies to circumvent the deleterious effects of high doses of the MHC-II neoAg on the antitumour response. Using this approach, we showed that the inhibition of antitumour efficacy by HDVax is due to the induction of cytolytic tumour antigen-specific Tr1 cells that kill cDC1s in vitro and in vivo. Thus, our work identifies a critical inhibitory function of a known CD4^+^ T cell subset that has not been generally associated with cancer immunotherapy or cancer immune escape. Our demonstration that Tr1 cells become detectable in progressively growing tumours as they become resistant to anti-PD1 argues strongly that the HDVax-induced cells are orthologues of those that arise during progressive tumour growth and, therefore, are physiologically relevant participants in cancer-related immunosuppression^38,52^. We then defined the mechanisms by which Tr1 cells are induced and demonstrated three alternative strategies to circumvent their activity in vivo.

We also showed that the phenotypic markers on Tr1 cells differ based on the anatomical sites from which they are isolated. Thus, whereas HDVax-induced Tr1-like cells in the spleen are differentially marked by expression of CD49b and LAG3 (ref. ^39^), the cells in TILs lack differential expression of these markers but still retain the capacity to produce IL-10, the signature cytokine of Tr1 cells. By contrast, LILRB4 expression clearly identifies mouse Tr1-like cells from both the spleen and the tumour microenvironment. However, whereas LILRB4 in mice is encoded by a single *Gb49b* gene, it belongs to a more complex family in humans comprising five different genes^53^. Efforts to define the human orthologue will be an essential goal of future work and could provide a tool to readily identify the presence of such cells in patients with cancer. This is important because HDVax-induced inhibitory Tr1 cells and effector LDVax-induced helper CD4^+^ T express similar levels of CD39, suggesting that other factors beyond CD39 are required to differentiate between CD4^+^ T cells that inhibit versus promote tumour rejection^54,55^.

Recent work by others has demonstrated that certain tumour antigens, termed inhibigens, can suppress antitumour efficacy of cancer vaccines and anti-PD1 (ref. ^56^), and that their inhibitory function was due to their inherent but undefined structural characteristics. This classification differentiates inhibigens from the MHC-II neoAgs presented in the current study, whose activating versus inhibitory properties are linked to their dosing. Our study raises the possibility that any MHC-II neoAg can be inhibigen-like when produced or released at elevated levels, thus broadening the inhibigen definition. It will also be essential in the future to explore whether the induction of Tr1 cells depends on the type of vaccine platform used (for example, peptide-based versus DNA-based versus RNA-based vaccines). Future studies will address these questions. Nevertheless, identifying Tr1 cells and their immunosuppressive biological function in cancer and providing three strategies to circumvent their inhibitory functions provide potentially novel targets for improving the effectiveness of cancer immunotherapies in general.

## Methods

### Mice

WT male 129S6 mice (for experiments involving T3 and F244 cells) and female C57BL/6 mice (for experiments involving 1956 cells) were purchased from Taconic Farms and Charles River, respectively. *Gzmb*^−/−^ on the C57BL/6 background and *Rag2*^−/−^ mice (on the 129S6 and C57BL/6 backgrounds) were bred and housed in our specific pathogen-free facility. cDC2-deficient (Δ1 + 2 + 3)^44–46^ and cDC1-deficient (IRF8Δ32)^44–46^ mice on the C57BL/6 background were obtained from K. Murphy (Washington University in St. Louis). *Lilrb4a* (encoding gp49b)-knockout mice were obtained from M. Colonna (Washington University in St. Louis) and were generated by the Genome Engineering and iPSC Center (GEiC) and the Transgenic, Knockout, and Micro-Injection Core, Department of Pathology and Immunology, at Washington University in St. Louis. A single guide RNA (cctttagttgcagctcatccata) and Cas9 proteins were electroporated into zygotes before being transferred into pseudo-pregnant B6/J female mice. The resulting founders were screened using next-generation sequencing (NGS). A male founder with a 21-nucleotide deletion in exon 4 was bred with B6/J female mice obtained from the Jackson Laboratory (JAX stock #000664) for one generation, and heterozygous F1 mice were intercrossed to generate knockouts. The integrity of the highly homologous gene *Lilrb4b* was confirmed by NGS. The absence of LILRB4A protein expression in blood monocytes and neutrophils was confirmed by flow cytometry using the H1.1 antibody^57^ clone (BioLegend). Lineage tracing eGFP mice on the C57BL/6 background were obtained from J. Kipnis (Washington University in St. Louis). Mice were used between 8 and 10 weeks of age. All in vivo experiments were performed in our specific pathogen-free facility using procedures approved by the AAALAC-accredited Animal Studies Committee of Washington University in St Louis and followed all relevant ethical regulations.

### Tumour transplantation

T3, F244 and 1956 are MCA-induced sarcoma cells that were previously generated in our laboratory in male 129S6 (T3 and F244 cell lines) and female C57BL/6 mice (1956 cell line). Tumour cells were cultured in RPMI medium (Hyclone) supplemented with 10% FCS (Hyclone). All cell lines used were negative for mycoplasma and other infectious agents. For tumour inoculation, tumour cells were harvested by trypsinization, washed three times with PBS and resuspended in PBS at a density of 10^7^ cells per millilitre. Then, 150 μl was injected subcutaneously into the rear flanks of syngeneic recipient mice. Tumour growth in individual mice was determined using callipers and expressed as the average of two perpendicular diameters. Mice were euthanized when the tumour diameter reached 20 mm in any direction.

### NeoAgs and vaccination protocol

T3 neoAgs were previously described^8,19,20^ and are listed in Supplementary Table 1. The peptide of HPV (DKCLKFYSKISEYRHY CYSLYGTTL) was used as an irrelevant non-sarcomas antigen. All peptides were purchased from Peptide 2.0 or Genscript with a specified purity of over 95%. Endotoxin levels in all peptides tested were below 0.5 EU ml^−1^ (Leinco). The indicated doses of peptide were mixed with 50 μg of polyinosinic–polycytidylic acid complexed with poly-l-lysine and carboxymethylcellulose (poly-ICLC; Oncovir) diluted in PBS. For therapeutic vaccination, tumour-bearing mice were injected intravenously or subcutaneously with the vaccines (200 μl) on days 6 and 17 post-tumour transplant. In experiments using the combination of vaccination and ICT, vaccines were administered intravenously on days 3 or 6 post-tumour transplantation. In the sorting experiments, 25 μg of anti-CD40 was mixed with the second dose of the vaccine to increase the yield of the antigen-specific CD4^+^ T cells.

### In vivo antibody treatment

For in vivo treatment, LPS-free and pathogen-free anti-PD1 (rat IgG2a, clone RMP1-14), anti-CTLA4 (mouse IgG2b, clone 9D9) or anti-4-1BB (rat IgG2a, clone 3H3) monoclonal antibodies were purchased from Leinco Technologies. Tumour-bearing mice were vaccinated on day 3 post-tumour implantation and then injected intraperitoneally with 200 μg of each antibody on days 3, 6 and 9. To assess the role of IL-10 in HDVax-induced inhibition, monoclonal antibodies to IL-10 (rat IgG1, clone JES5-2A5) and IL-10R (rat IgG1, clone 1b1.3A) were purchased from Bioxcell and injected with HDVax. For anti-IL-10 blockade, mice were treated with 250 μg 1 day before tumour implantation, followed by treatment every 3 days for the duration of the experiment. For anti-IL-10R blockade, mice were treated with 1 mg per mouse every 7 days, starting 1 day before tumour injection. Neutralizing but not depleting hamster monoclonal antibody specific for LILRB4 (clone# 2F1.F9.E6) was provided by N. Sharma and J. Allison (University of Texas (MD Anderson Cancer Center)), and mice were treated with 250 μg on days 3, 6 and 9 post-tumour inoculation.

### Tetramer staining

Tetramer staining for mLama4-specific CD8^+^ T cells and mItgb1-specific CD4^+^ T cells was performed as previously described^8,21^. TILs or splenocytes (1–4 million) in 50 μl FACS buffer were incubated with PE/APC-labelled mLama4 peptide–H-2K^b^ tetramer at 37 °C for 20 min. Then, an antibody master mix consisting of CD90.2, CD4, CD11b and live/dead dye (NIR) in 50 μl FACS buffer was added to each well and incubated for 30 min at 4 °C. Tetramers were obtained from the Bursky Center Immunomonitoring Laboratory at Washington University School of Medicine in St. Louis.

### Flow cytometry antibodies

Flow antibodies were: IFNγ (XMG1.2; 1:100; 505808), TNF (MP6-XT22; 1:100; 506323), CD200 (OX-90; 1:200; 123820), PD1 (29F.1A12; 1:200; 135228), CCL5 (2E9/CCL5; 1:500; 149106), GZMB (QA16A02; 1:50; 372216), TIM3 (RMT3-23; 1:200; 119723), CD25 (PC61; 1:100; 102036), CD154 (MR1; 1:100; 106506), CD152 (UC10-4B9; 1:100; 106318), CD4 (RM4-5; 1:500), IL-2 (Jes6-5H4; 1:50; 503826), LILRB4 (H1.1; 1:200; 144904), CD11b (M1/70; 1:800; 101226), XCR1 (ZET; 1:100; 148206), MHC-II (M5/114.15.2; 1:1000; 107641), CD11c (N418; 1:200; 117336), CD172a (P84; 1:500; 144008), Zombie NIR fixable viability dye (1:500; 423106), CD86 (GL-1; 1:200; 105042), CD80 (16-10A1; 1:200; 104712) and CD40 (FGK45; 1:100; 157506) all from BioLegend; CD90.2 (53-2.1; 1:800; 565257), CD45 (HI30; 1:800; 563791), CD39 (Y23-1185; 1:400; 567105), CD153 (RM153; 1:200; 740751), CD70 (FR70; 1:100; 740741) and CD8 (53.6.7; 1:200; 564920) from BD Biosciences; or FOXP3 (Fjk-16a; 1:50; 11-5773-82) and SEMA4a (5E3; 1:50; 46-9753-41) from eBioSciences. Foxp3/Transcription factor Staining kit (00-5523-00, Thermo Fisher) was used to stain FOXP3 and other intracellular proteins according to the manufacturing protocol. BD FACSDIVA software V9.1 on Symphony 3, LSRFortessaX20 or ARIA-II was used for collecting the flow cytometry data and for sorting, respectively. Data were analysed using FlowJo software v10.10.

### Multiplex assay and antibody ELISA

TILs were harvested 7 days post-vaccination, stained with mItgb1–I-A^b^ tetramer prepared by the Immunomonitoring Laboratory core at Washington University in St. Louis using mItgb1 peptide covalently attached to the I-A^b^ β-chain, and total mItgb1-specific CD4^+^ T cells (Fig. 2c) or cells populating different clusters (Fig. 3c) were sorted by flow cytometry. Sorted cells (*n* = 200,000) were stimulated in a serum-free medium with 10^6^ irradiated splenocytes (isolated from naive mice) pulsed with 1 μg ml^−1^ mItgb1 SLP. Following a 72-h incubation, secretion of multiple cytokines was measured using a flow-based customized ProcartaPlex cytokine panel (Luminex Technologies) following the manufacturer’s protocol.

To determine antibody responses to the mItgb1 peptide, ELISA plates were coated with 10 μg ml^−1^ of either mItgb1 or irrelevant SLP at 4 °C overnight. Plates were washed and blocked with 4% goat serum for 2 h at room temperature, and then different dilutions of serum from LDVax or HDVax mice were added for 2–6 h. Plates were washed again and incubated with peroxidase-conjugated goat antibody to total mouse IgG (H + L) (115-035-003, Jackson ImmunoResearch) or different isotype-specific secondary reagents conjugated with horseradish peroxidase for 2 h at room temperature, followed by sequential addition of tetramethylbenzidine substrate (TMB). The reaction was stopped by acidification, and the optical density of each well was measured at 405 nm.

### Adoptive transfer experiments

For adoptive transfer experiments into *Rag2*^–/–^ mice, WT mice were vaccinated with LDVax, and 9 days later, 5 million total T cells were transferred into *Rag2*^−/−^ mice that were injected with 2 × 10^5^ tumour cells 1 day earlier. To isolate mItgb1-specific CD4^+^ T cells from HDVax mice, TILs and spleens were harvested from HDVax-treated mice, and CD4^+^ T cells were enriched using the CD4^+^ T cell isolation kit from Miltenyi (130-117-043) stained with mItgb1 tetramer, and CD25-negative mItgb1-specific CD4^+^ T cells were purified by flow cytometry. Half a million mItgb1-specific CD4^+^ T cells were injected intratumourally (in 50 μl) into T3 tumour-bearing *Rag2*^−/−^ mice.

For sorting and adoptive transfer of individual clusters, CD4^+^ T cells were enriched by positive bead selection and stained with mItgb1–I-Ab^+^ tetramers, CD25, CD200, CD153 and CD39. T_reg_ cells were sorted based on the positive expression of CD25, and non-cluster 3/5 cells were defined by the negative expression of CD25 and positive expression of CD200 and/or CD153. Cluster 3 cells were sorted based on the negative expression of CD25, CD200 and CD153 and the positive expression of CD39. An equal number of cells (2 × 10^5^ cells) sorted from these clusters were resuspended in 50 μl PBS and were injected intratumourally. To sort for LILRB4-expressing and SEMA4-expressing cells, CD25, CD200 and CD39 were used to pregate on cluster 3 cells, and 1 × 10^5^ cells were injected into T3 tumour-bearing *Rag2*^−/−^ mice that also received T_reg_ cell-depleted total T cells from LDVax mice.

### Intracellular cytokine and CD40L staining

Splenocytes (10^5^) harvested from naive mice were irradiated (30 Gy) and pulsed with 1 μg ml^−1^ peptide, and TILs (1–2 × 10^6^) were subsequently added, and the cell suspension was incubated at 37 °C. GolgiPlug (BD Biosciences) was added 1 h later and incubated for another 4 h. Cells were stained for different surface markers, including the live/dead marker (NIR), then permeabilized using the intracellular permeabilization kit (BD Biosciences), followed by staining for IFNγ, TNF and CD40L (clone; SA047C3; 157010, BioLegend).

### Ex vivo antigen presentation assay

Naive WT mice were intravenously vaccinated with HDVax consisting of 15 μg of mItgb1 SLP and 50 μg poly-ICLC (HDVax). At the specified time point, spleens and lymph nodes were harvested and digested in the presence of collagenase. APCs were FACS sorted into four different subpopulations: cDC1 (MHCII^+^CD11c^+^XCR1^+^), cDC2 (MHCII^+^CD11c^+^CD172a^+^) MHCII^+^CD11c^−^ and MHCII^−^CD11c^−^. Sorted APCs (5 × 10^3^) were incubated with 5 × 10^4^ mItgb1-specific hybridoma cells^8^ in IL-2 precoated ELISPOT plates (IMMUNOSPOT). Twenty-four hours later, plates were developed following the manufacturer’s protocol, and IL-2 spots were quantified using a CTL ImmunoSpot S6 machine.

### In vitro dendritic cell killing assay

To isolate dendritic cells, spleens were harvested from naive WT mice and disrupted by collagenase digestion. CD11c^+^ cells were enriched using a positive selection kit (130-125-835, Miltney Biotec). Enriched CD11c^+^ cells were pulsed with mItgb1 SLP at 1 μg ml^−1^ and incubated with 1 × 10^4^ tumour-specific CD4^+^ T cells at a 1:1 ratio for 12 h. Cells were washed twice with PBS, stained with MHC-II, CD11c, XCR1, CD172a and Zombie NIR dye to determine the frequency of NIR in cDC1s and cDC2s using flow cytometry.

### CD8–IL-2

CD8–IL-2 was provided by Asher Biotherapeutics^41^. CD8–IL-2 was generated via fusion of an IL-2 mutein that does not bind to IL-2Rα and displays significantly reduced binding to IL-2Rβγ. This mutein was then coupled to a monovalent mouserized anti-mouse CD8 antibody. Monovalent IL-2 coupling was achieved using bispecific charge pair technology, and FcγR binding was abolished via mutating the FcγR binding region of an anti-CD8 antibody. CD8–IL-2 was expressed in HEK293 cells and purified via protein A affinity chromatography, followed by ion-exchange chromatography and then size-exclusion chromatography. Therapeutic doses (1 mg kg^−1^) of CD8–IL-2 induced significant antitumour activity (approximately 80% response rate) against 8-day-established T3 tumours when they became insensitive to anti-PD1 therapy. For the current study, CD8–IL-2 was administered at a subtherapeutic dose of 0.3 mg kg^−1^ diluted in PBS and injected intraperitoneally. WT IL-2 (202-IL/CF) was purchased from (R&D Systems). Each mouse was treated with 25,000 IU in PBS injected intraperitoneally daily for 5 days.

### scRNA-seq analysis

#### UMAP clustering and separation of total and antigen-specific cells

T3 tumour-bearing mice were treated with HDVax, LDVax or PBS 6 days post-tumour transplantation. Seven days later, single-cell suspensions were prepared from TILs (pooled from seven mice for each group). Total CD4^+^ T cells were enriched using a CD4^+^ T cell-positive selection kit (Miltenyi). Enriched CD4^+^ T cells were split into two fractions. One fraction was labelled with TCRβ (TotalSeq-C0120 anti-mouse TCRβ chain antibody, 109259, BioLegend) and used as a source of total CD4^+^ T cells. The other fraction was labelled with CD90.2 (TotalSeq-C0075 anti-mouse CD90.2 antibody, 105353, BioLegend) and used to isolate mItgb1-specific cells based on mItgb1–I-A^b^ tetramer staining and flow sorting. The two fractions (total and antigen-specific CD4^+^ T cells) were mixed at a 1:1 ratio and submitted to the Genome Institute at Washington University to generate 10X libraries using 10X 5′ v2 single-cell RNA-seq. Alignment, barcode assignment and unique molecular identifier counting with Cell Ranger (v6.1.1) were used to prepare count matrices for the gene expression library using the mouse genome (GRCm38) as a reference^58^. Barcodes in all samples representing low-quality cells were filtered out using the standard knee-inflection strategy available in Cell Ranger. For downstream analysis, the Seurat package (v4.0.4) was used; genes expressed in fewer than three cells were additionally filtered out from expression matrices, and cells that contained fewer than 200 expressed genes were removed. The mitochondrial gene fraction was calculated for all cells, and cells with a mitochondrial fraction more than the highest confidence interval for scaled mitochondrial percentage were filtered out. Each sample was normalized using the SCTransform function with mitochondrial content as a variable to regress out in a second non-regularized linear regression. For integration purposes, variable genes across the samples were identified by the SelectIntegrationFeatures function with a number of features equal to 2,000. Then, the object was prepared for integration (PrepSCTIntegration function), the anchors were found (FindIntegrationAnchors function) and the samples were integrated into the whole object (IntegrateData function)^59^. Principal component analysis (PCA) was used for dimensionality reduction, and the first 20 principal components were used further to generate UMAP dimensionality reduction by the RunUMAP function. The clustering procedure was performed by FindNeighbors and FindClusters with a range of resolutions (from 0.2 to 1.0, with 0.2 as a step) and the first 20 principal components as input. Antibody-derived tags (ADT) data were normalized by a centred log-ratio transformation method, scaled and transformed to its own PCA space. Finally, cells were defined as mItgb1-specific cells and total cells (predominantly mItgb1-nonspecific cells) based on the scaled value of corresponding ADTs. Each object was iteratively cleaned for doublets as well as low-quality cells.

### TCR data analyses

TCR data were aligned to the reference mouse genome GRCm38 and counted using Cell Ranger (v6.1.1) vdj workflow^58^. The Seurat object was converted to the h5ad format, and cells that passed quality control for gene expression and TCR levels were integrated into the objects (mItgb1-specific cells and total cells). Downstream analyses, including clonotype expansion analysis and estimation of used VDJ gene pairings, were executed using the scirpy toolkit. Proportions of shared and unique clonotypes with CD4^+^FOXP3^+^ T_reg_ cells (cluster 5) were counted. TCR analyses were done using the Immunarch package.

### Bulk RNA-seq

Three cohorts (*n* = 5) of T3 tumour-bearing mice were vaccinated with HDVax, and 7 days later CD4^+^ T cells were enriched from TILs using the Miltenyi mouse CD4^+^ T cells isolation kit, stained with the mItgb1–I-A^b^ tetramer and then sorted into three replicates of CD25^+^ (T_reg_ cells), CD25^−^CD39^−^CD200^+^ (non-T_reg_ cells/non-cluster 3) and CD25^+^CD200^−^CD39^+^ (cluster 3). Cells were lysed, and mRNA was extracted using Nucleospin RNA Plus (740990.50, TaKaRa). RNA was sequenced by the Washington University Genome Institute. In brief, total RNA integrity was determined using Agilent Bioanalyzer. Library preparation was performed with 10 ng of total RNA with a Bioanalyzer RNA integrity number (RIN) score greater than 8.0. double-stranded DNA (ds-cDNA) was prepared using the SMARTer Ultra Low RNA kit for Illumina sequencing (740990.50, TaKaRa) per the manufacturer’s protocol. cDNA was fragmented using a Covaris E220 sonicator using peak incident power of 18, duty factor of 20%, and cycles per burst of 50 for 120 s. cDNA was blunt-ended, had an A base added to the 3′ ends, and then had Illumina sequencing adapters ligated to the ends. Ligated fragments were then amplified for 12–14 cycles using primers incorporating unique dual index tags. Fragments were sequenced on an Illumina NovaSeq-6000 using paired-end reads extending 150 bases. For analysis, raw data were aligned and counted to the GRCm38.101 reference genome by the Dragen workflow of the GTAC@MGI service. For downstream analysis, 12,000 genes with the highest number of counts across the samples were selected. All samples were normalized by regularized log transformation (rlogTransformation function) and variance stabilizing transformation (vst function), which are accomplished in the DESeq2 (v1.30.1) package. PCA was run based on the vst output, and no outliers were identified. log_2_ fold change values were shrunk for all comparisons (CD25 versus CD39, and CD200 versus CD39) by the lfcShrink function, and its output was used for volcano plot construction^60^.

### Gene signature analysis

To evaluate the significance of enrichment for highly expressed genes within cluster 3, we identified the top 200 differentially expressed genes from a bulk RNA-seq dataset (Supplementary Table 2). Subsequently, we conducted a rigorous analysis of differential gene expression using the limma package on normalized count data obtained from publicly available datasets. Specifically, we performed the following pairwise comparisons.

For the cytotoxic CD4^+^ T cells, data from GSE141540 were analysed, and a comparison of T_H_ cell-ctx versus T_H_ cell was performed. For the Tr1 gene signature, GSE158703 and GSE139990 were obtained, and a comparison of Tr1–IL-10^Pos^ versus T_H_17–IL-10^Neg^ or Tr1 versus naive was performed, respectively.

For the T_reg_ and T_FH_ gene signature, GSE68242 or GSE140187 were obtained, and a comparison of FOXP3 versus other or lymph node WT T_FH_ versus lymph node naive was performed, respectively. For the chronically stimulated exhausted CD4^+^ T cells, data from GSE30431 were analysed, and a comparison of CD4 D30 chronic versus CD4 D30 acute was performed. We used GSEA to ascertain the significance of enrichment for the gene signature associated with cluster 3. This method evaluates whether cluster 3-predefined gene signatures are statistically overrepresented or underrepresented within the differentially expressed gene lists derived from the publicly available bulk RNA-seq datasets for each CD4^+^ T cell subtype.

To determine the frequency of Tr1-like cells in patients with cancer, we first subset the quality-controlled cells from each manuscript down to CD4^+^ T cells. After this, we used AUCell^61^ to determine which cells match the human Tr1 gene set. Following this, we determined the proportion of the Tr1 cells within the CD4^+^ T cell population of each patient. We tested for differences in Tr1 cell proportions between responders and non-responders using Student’s *t*-tests without assuming equal variances. There is a significant difference in the proportion of Tr1 cells between responders and non-responders from the Sade-Feldman et al. dataset (*P* = 0.002, *t* = 4.98, d.f. = 6.11). There was no significant difference in the Awad et al. dataset (*P* = 0.55).

### GSEA for the IL-2 pathway

The FindMarkers function was used to find genes specific to both LDVax and HDVax samples. The average log_2_ fold change values were used as an input in GSEA implemented in the fgsea R package (v1.16.0). Forty-three genes associated with the IL-2 signalling pathway were used for the gene set enrichment plot^62^. For violin plots, the summary expression of IL-2 signalling genes was calculated using the sctransform (SCT) assay (data slot), scaled and divided by the number of genes in the pathway.

### Statistics

GraphPad Prism software (v10.2.2) was used to perform all statistical analyses. Significance was determined using one-way ANOVA with multiple comparisons corrected with Tukey’s method unless otherwise stated in the figure legends.

### Reporting summary

Further information on research design is available in the Nature Portfolio Reporting Summary linked to this article.

## Online content

Any methods, additional references, Nature Portfolio reporting summaries, source data, extended data, supplementary information, acknowledgements, peer review information; details of author contributions and competing interests; and statements of data and code availability are available at 10.1038/s41586-024-07752-y.

### Supplementary information

Supplementary Information

Reporting Summary

### Source data

Source Data Fig. 1

Source Data Fig. 2

Source Data Fig. 3

Source Data Fig. 4

Source Data Fig. 5

Source Data Extended Data Fig. 1

Source Data Extended Data Fig. 2

Source Data Extended Data Fig. 3

Source Data Extended Data Fig. 4

Source Data Extended Data Fig. 5

Source Data Extended Data Fig. 6

Source Data Extended Data Fig. 7

Source Data Extended Data Fig. 8

Source Data Extended Data Fig. 9

Source Data Extended Data Fig. 10

## Data Availability

Bulk RNA-seq and scRNA-seq data were deposited using the accession number GSE268302; GSE268300 (bulk RNA-seq) or GSE268301 (scRNA-seq) can be used to access raw data. The pipelines with code to process and analyse the bulk RNA-seq and scRNA-seq data are available at https://github.com/vdsukhov/neoantigen_vaccines_2024. Source data are provided with this paper.
