# Supplementary material for: Neoantigen-specific cytotoxic Tr1 CD4 T cells suppress cancer immunotherapy
Source: Nature. 2024 Jul 24;632(8023):182–91. doi: 10.1038/s41586-024-07752-y (PMC11291290; doi:10.1038/s41586-024-07752-y)
Supplement: Supplementary file 1 — Supplementary Information [file 41586_2024_7752_MOESM1_ESM.pdf]

nature portfolio

https://doi.org/10.1038/s41586-024-07752-y

Supplementary information

# Neoantigen-specific cytotoxic Tr1 CD4 T cells suppress cancer immunotherapy

In the format provided by the authors and unedited

# Neoantigen-Specific Cytotoxic Tr1 CD4 T Cells Suppress Cancer Immunotherapy

Hussein Sultan[1,2], Yoshiko Takeuchi[1,2], Jeffrey P. Ward[3], Naveen Sharma[4], Tian-Tian Liu[1], Vladimir Sukhov[1], Maria Firulyova[5] , Yuang Song[1,2], Samuel Ameh[1,2], Simone Brioschi[1], Darya Khantakova[1], Cora D. Arthur[1,2], J. Michael White[1], Heather Kohlmiller[1,2], Andres M. Salazar[6], Robert Burns[7], Helio A. Costa[7], Kelly D. Moynihan[8], Yik Andy Yeung[8], Ivana Djuretic[8], Ton N. Schumacher[9], Kathleen C. F. Sheehan[1,2], Marco Colonna[1], James P. Allison[4,10], Kenneth M. Murphy[1], Maxim N. Artyomov[1], and Robert D. Schreiber[1,2,10;11]

**Affiliations**

[1]Department of Pathology and Immunology, Washington University School of Medicine, St. Louis, MO, [2]The Andrew M. and Jane M. Bursky Center for Human Immunology and Immunotherapy Programs, Washington University School of Medicine, St. Louis, MO, [3]Division of Oncology, Department of Medicine, Washington University School of Medicine, St. Louis, MO, [4]Department of Immunology, University of Texas MD Anderson Cancer Center, Houston, TX,[5]Almazov National Medical Research Centre, St.Petersburg, Russia, [6]Oncovir, Inc., Washington DC, [7]Natera, Inc., Austin, TX, [8]Asher Biotherapeutics, South San Francisco, CA, [9]Netherlands Cancer Institute, Oncode Institute, Amsterdam, Leiden University, Leiden, Netherlands, [10]The Parker Institute for Cancer Immunotherapy, San Francisco, CA, USA, [11]e-mail: rdschreiber@wustl.edu

**Supplemental Table 1[*].**

| **Sarcoma line** | **Mouse strain** | **Sex** | **MHC allele** | **Gene** | **SLP used in the vaccine** |
|---|---|---|---|---|---|
| **T3** | 129S6 | Male | MHC-I (H-2K$^b$) | ***Lama4***<br>***Alg8*** | KISFFDGFEVGFNFRTLQPNGLLFYYT<br>AVGITYTWTRLYASVLTGSL |
| | | | MHC-II (I-A$^b$) | ***Itgb1*** | DDCWFYFTYSVNGYNEAIVHVVETPDCP |
| **F244** | 129S6 | Male | MHC-I (H-2K$^b$) | ***Pex14*** | YGALAIIMAGIAFAFHQLYKRYLLPLIL |
| | | | MHC-II (I-A$^b$) | ***Plec*** | LFQAMKKDLIVRYHGVRLLEAQIAT |
| **1956** | C57BL/6N | Female | MHC-I (H-2K$^b$) | ***Psmd6*** | AVRENNIAPYYEALCKSLDWQMDVDLL |
| | | | MHC-II (I-A$^b$) | ***Cs*** | TEEQVSWLSREWAKSAALPSHVVTMLDN |

**Supplemntal Table 1.** The synthetic long peptide sequences used in the vaccination studies. The binding core is underlined.

**Supplemental Table 2:**

| gene_symbol | logFC | AveExpr | t | P.Value | adj.P.Val | B |
|---|---|---|---|---|---|---|
| Gzmb | 7.79577848 | 6.20656977 | 38.3530939 | 6.70E-17 | 4.35E-13 | 28.6459567 |
| Ccl5 | 6.37668419 | 10.4779208 | 37.4823444 | 9.56E-17 | 4.35E-13 | 28.0303607 |
| Ccr2 | 5.36149305 | 5.37582485 | 31.7108801 | 1.27E-15 | 3.46E-12 | 25.9475391 |
| Ccl4 | 5.9192673 | 4.39843145 | 30.5417271 | 2.26E-15 | 5.15E-12 | 25.252574 |
| Cxcr6 | 4.16665655 | 9.31136991 | 27.5823146 | 1.08E-14 | 1.81E-11 | 23.9127833 |
| Cx3cr1 | 5.58745227 | 3.45148977 | 27.41215 | 1.19E-14 | 1.81E-11 | 23.495452 |
| Ccr5 | 4.95203584 | 4.11218566 | 26.863922 | 1.62E-14 | 2.22E-11 | 23.4080236 |
| Sema4a | 4.01485796 | 4.96365667 | 25.5906748 | 3.42E-14 | 3.83E-11 | 22.8320927 |
| Lilr4b | 3.65168793 | 5.09377661 | 23.648104 | 1.14E-13 | 1.04E-10 | 21.6832626 |
| Chsy1 | 3.33859925 | 5.52356244 | 23.5023243 | 1.25E-13 | 1.07E-10 | 21.6066952 |
| Lilrb4a | 3.22837184 | 5.23095709 | 23.4027229 | 1.34E-13 | 1.08E-10 | 21.5348027 |
| Serpina3f | 4.11857904 | 4.77752504 | 22.6056464 | 2.27E-13 | 1.72E-10 | 21.002497 |
| Prdm1 | 3.84723076 | 4.05593633 | 22.5221114 | 2.40E-13 | 1.73E-10 | 20.8891294 |
| Adam8 | 3.68530006 | 5.25672282 | 22.2418791 | 2.90E-13 | 1.78E-10 | 20.780104 |
| Gas7 | 4.17706937 | 4.74310449 | 22.2378928 | 2.91E-13 | 1.78E-10 | 20.7589356 |
| Matn2 | 3.34521324 | 4.93090018 | 22.1934226 | 3.00E-13 | 1.78E-10 | 20.7376913 |
| Csf1 | 4.52456914 | 4.1236649 | 21.946946 | 3.56E-13 | 2.02E-10 | 20.521677 |
| Fgl2 | 5.59858592 | 2.08122184 | 21.532432 | 4.75E-13 | 2.53E-10 | 19.737406 |
| F2r | 3.07983266 | 5.36997056 | 21.4437754 | 5.05E-13 | 2.53E-10 | 20.2367955 |
| Sytl2 | 4.50806623 | 3.68753785 | 20.85665 | 7.70E-13 | 3.63E-10 | 19.736619 |
| Entpd1 | 4.51005832 | 4.66211172 | 20.7379865 | 8.39E-13 | 3.82E-10 | 19.7231286 |
| Il10 | 5.96755197 | 3.29378388 | 20.6189685 | 9.16E-13 | 3.98E-10 | 19.513229 |
| Tmprss13 | 4.04427117 | 3.44690461 | 20.5842537 | 9.39E-13 | 3.98E-10 | 19.5145997 |
| Prf1 | 4.21509335 | 4.67904196 | 20.5538026 | 9.60E-13 | 3.98E-10 | 19.5914019 |
| Ifngr1 | 2.95875415 | 6.7046412 | 20.4130737 | 1.07E-12 | 4.14E-10 | 19.4988427 |
| Serpina3g | 2.82024228 | 6.61015104 | 20.341787 | 1.12E-12 | 4.14E-10 | 19.4460221 |
| Ccl3 | 5.9559615 | 1.71363753 | 20.3302471 | 1.13E-12 | 4.14E-10 | 18.8289384 |
| Pik3ap1 | 5.54598274 | 2.35476735 | 20.3073509 | 1.15E-12 | 4.14E-10 | 19.0669082 |
| Tmem37 | 4.01322046 | 3.70537292 | 20.0802304 | 1.37E-12 | 4.78E-10 | 19.1909706 |
| Metrnl | 5.81401777 | 1.53743197 | 19.7873414 | 1.70E-12 | 5.82E-10 | 18.4000307 |
| Kcnk5 | 3.08996338 | 5.37159179 | 18.820205 | 3.62E-12 | 1.18E-09 | 18.278 |
| Pvrig | 4.33963367 | 2.63656695 | 18.6489051 | 4.16E-12 | 1.32E-09 | 17.9736953 |
| F2rl2 | 6.50675757 | 1.88093549 | 18.5839318 | 4.38E-12 | 1.34E-09 | 17.7210582 |
| Il12rb2 | 3.83534147 | 3.00201045 | 18.1273427 | 6.36E-12 | 1.81E-09 | 17.6372865 |
| Tm6sf1 | 5.00916387 | 1.89459447 | 17.5486841 | 1.03E-11 | 2.77E-09 | 16.9572333 |
| Il2ra | 4.21092001 | 2.67994769 | 17.039517 | 1.60E-11 | 4.06E-09 | 16.7150539 |
| Ifi214 | 2.37222728 | 5.87277348 | 16.5124884 | 2.56E-11 | 6.03E-09 | 16.2977329 |
| Asb2 | 2.67313816 | 6.08816559 | 16.4001768 | 2.83E-11 | 6.55E-09 | 16.1918711 |
| Arnt2 | 3.41618314 | 2.78405558 | 16.2166394 | 3.35E-11 | 7.62E-09 | 16.025623 |
| Plek | 2.68841103 | 6.26756989 | 15.911122 | 4.43E-11 | 9.61E-09 | 15.7334148 |

| | | | | | | |
|---|---|---|---|---|---|---|
| Slamf1 | 2.26907491 | 6.09963603 | 15.7190605 | 5.30E-11 | 1.13E-08 | 15.5518882 |
| Cish | 3.01034924 | 4.89205644 | 15.6271994 | 5.78E-11 | 1.22E-08 | 15.4972224 |
| Acsbg1 | 2.25338654 | 5.10886351 | 15.5405172 | 6.28E-11 | 1.28E-08 | 15.4058874 |
| 1700017B05Rik | 2.40360946 | 5.03614851 | 15.4245069 | 7.01E-11 | 1.39E-08 | 15.2964154 |
| L1cam | 2.3762347 | 5.1563049 | 15.2853618 | 8.01E-11 | 1.52E-08 | 15.1561173 |
| Myadm | 2.52370697 | 4.40047225 | 15.2651379 | 8.17E-11 | 1.53E-08 | 15.1664084 |
| Plac8 | 4.69400442 | 1.97578018 | 15.2269626 | 8.48E-11 | 1.56E-08 | 15.0363852 |
| Gpld1 | 4.41018084 | 1.42172622 | 14.9952823 | 1.06E-10 | 1.91E-08 | 14.7161919 |
| Foxp3 | 6.67593825 | 0.49768022 | 14.95615 | 1.10E-10 | 1.93E-08 | 14.3576579 |
| Agpat4 | 2.59736274 | 5.30698847 | 14.8835062 | 1.19E-10 | 2.03E-08 | 14.7513372 |
| Mmp9 | 7.88491289 | -1.4754909 | 14.7845823 | 1.31E-10 | 2.18E-08 | 12.7964156 |
| Ppp3ca | 3.22688679 | 3.23006369 | 14.7500551 | 1.35E-10 | 2.23E-08 | 14.6857181 |
| Padi2 | 4.10411701 | 2.21076553 | 14.7279411 | 1.38E-10 | 2.25E-08 | 14.6056779 |
| Ifi206 | 2.71685655 | 4.29827892 | 14.7094669 | 1.41E-10 | 2.26E-08 | 14.6206359 |
| Lag3 | 2.12640653 | 7.93236832 | 14.5975333 | 1.58E-10 | 2.42E-08 | 14.4526452 |
| Nkg7 | 2.44658558 | 10.5249798 | 14.5545576 | 1.64E-10 | 2.50E-08 | 14.4526172 |
| St6galnac2 | 1.85273843 | 6.65264226 | 14.3306706 | 2.06E-10 | 3.06E-08 | 14.1588807 |
| Rnase4 | 2.18340807 | 5.80110574 | 14.3140802 | 2.10E-10 | 3.08E-08 | 14.1507536 |
| Cdkn1a | 2.98174651 | 3.36231689 | 14.2605001 | 2.22E-10 | 3.22E-08 | 14.1956247 |
| Osgin1 | 2.62082498 | 3.65248109 | 14.1305943 | 2.53E-10 | 3.64E-08 | 14.0552096 |
| Arsb | 2.59730032 | 4.24929628 | 14.100811 | 2.61E-10 | 3.72E-08 | 13.9981948 |
| Eif4e3 | 2.21941598 | 4.75603422 | 14.0596736 | 2.73E-10 | 3.73E-08 | 13.9280537 |
| Fasl | 2.25849689 | 5.22992118 | 14.0457367 | 2.77E-10 | 3.73E-08 | 13.8891954 |
| Gm6637 | 3.87506899 | 2.88140981 | 13.9499856 | 3.05E-10 | 4.01E-08 | 13.8777268 |
| Heatr9 | 6.11502225 | 1.26886884 | 13.6707745 | 4.10E-10 | 5.13E-08 | 13.4399031 |
| Ctla2a | 2.48041252 | 3.46835009 | 13.627414 | 4.29E-10 | 5.28E-08 | 13.5330789 |
| Adgrg1 | 7.43120285 | -1.1598239 | 13.5129827 | 4.85E-10 | 5.86E-08 | 12.1394623 |
| Slc52a3 | 3.43453099 | 2.32292711 | 13.463362 | 5.11E-10 | 6.07E-08 | 13.3545326 |
| Lgals3 | 2.05311507 | 8.0858388 | 13.2632554 | 6.35E-10 | 7.30E-08 | 13.0268422 |
| Aopep | 1.99637339 | 5.5489608 | 13.2435467 | 6.49E-10 | 7.38E-08 | 13.0004254 |
| Ctla4 | 1.72929148 | 7.18086381 | 13.1459332 | 7.22E-10 | 8.08E-08 | 12.8762072 |
| Slc16a6 | 3.08902575 | 3.23107822 | 13.0455683 | 8.06E-10 | 8.88E-08 | 12.9097536 |
| Kctd12 | 4.65935369 | 0.89277497 | 13.0332062 | 8.17E-10 | 8.93E-08 | 12.721717 |
| Tnfrsf1b | 1.94785825 | 7.11238332 | 12.8195184 | 1.04E-09 | 1.08E-07 | 12.5023408 |
| Ifit1bl1 | 2.0679745 | 4.95813864 | 12.8007924 | 1.06E-09 | 1.09E-07 | 12.5284189 |
| Lamb3 | 2.12328366 | 3.99149237 | 12.7947653 | 1.07E-09 | 1.09E-07 | 12.5864887 |
| Coro2a | 2.00191487 | 5.22396112 | 12.7606232 | 1.11E-09 | 1.13E-07 | 12.4663744 |
| Laptm4b | 2.92868671 | 2.68975774 | 12.6867476 | 1.20E-09 | 1.22E-07 | 12.5221371 |
| Jaml | 1.97582318 | 4.7304303 | 12.5852296 | 1.35E-09 | 1.36E-07 | 12.2936186 |
| Atp2b4 | 2.35854992 | 5.09596689 | 12.5796325 | 1.36E-09 | 1.36E-07 | 12.2632733 |
| Sell | 2.09085667 | 6.19663872 | 12.4697445 | 1.54E-09 | 1.51E-07 | 12.0915001 |
| Gimap7 | 1.86134353 | 8.69090189 | 12.3962776 | 1.68E-09 | 1.62E-07 | 12.0429238 |

| | | | | | | |
|---|---|---|---|---|---|---|
| Dapk2 | 2.83508611 | 2.72070164 | 12.3895716 | 1.69E-09 | 1.63E-07 | 12.1852494 |
| 5830411N06Rik | 7.34001258 | -1.2393906 | 12.3655955 | 1.74E-09 | 1.66E-07 | 11.1204772 |
| Hip1 | 2.08800673 | 5.09132639 | 12.2676039 | 1.95E-09 | 1.83E-07 | 11.8945554 |
| Glrx | 1.81415972 | 8.25504068 | 12.2654803 | 1.95E-09 | 1.83E-07 | 11.8762404 |
| Epas1 | 3.18367635 | 2.2555568 | 12.085397 | 2.41E-09 | 2.24E-07 | 11.8367463 |
| Gbp3 | 1.59837363 | 6.35826962 | 12.0632141 | 2.47E-09 | 2.28E-07 | 11.6018118 |
| Ube2l6 | 2.00172614 | 5.50857661 | 11.9866932 | 2.71E-09 | 2.48E-07 | 11.531642 |
| Pglyrp1 | 1.7387295 | 6.88566864 | 11.8914206 | 3.03E-09 | 2.72E-07 | 11.3922948 |
| 1500009L16Rik | 4.15813747 | 2.31181096 | 11.8573016 | 3.16E-09 | 2.80E-07 | 11.570444 |
| Galnt2 | 1.84386554 | 5.75993558 | 11.686182 | 3.88E-09 | 3.37E-07 | 11.1495837 |
| Hid1 | 2.50403793 | 2.73649457 | 11.6773057 | 3.92E-09 | 3.39E-07 | 11.346532 |
| Gbp6 | 1.68667811 | 6.15187152 | 11.5901075 | 4.36E-09 | 3.70E-07 | 11.0187326 |
| Cyth4 | 1.67284533 | 7.88273532 | 11.5823088 | 4.40E-09 | 3.71E-07 | 11.0280033 |
| Ldlr | 2.23392071 | 3.77479499 | 11.5654981 | 4.49E-09 | 3.76E-07 | 11.139762 |
| Neb | 5.78675614 | 0.53728125 | 11.4784502 | 4.99E-09 | 4.13E-07 | 10.9773699 |
| Pdgfb | 2.61650138 | 2.36649227 | 11.4464772 | 5.19E-09 | 4.27E-07 | 11.076962 |
| Ddx28 | 1.71284923 | 5.8825719 | 11.4020162 | 5.48E-09 | 4.49E-07 | 10.7867988 |
| Gm33782 | 4.13286685 | 0.25987338 | 11.3942992 | 5.54E-09 | 4.50E-07 | 10.8174969 |
| Dennd5a | 2.31601839 | 3.15477414 | 11.3787728 | 5.64E-09 | 4.56E-07 | 10.9565307 |
| D16Ertd472e | 1.5483859 | 5.93489197 | 11.2870295 | 6.32E-09 | 5.02E-07 | 10.6379733 |
| Txndc5 | 1.74811471 | 5.1898307 | 11.2554215 | 6.58E-09 | 5.19E-07 | 10.6332021 |
| Aplp2 | 1.67128104 | 6.17984202 | 11.1917759 | 7.12E-09 | 5.56E-07 | 10.5096174 |
| Clic4 | 2.22282807 | 4.16472221 | 11.1081218 | 7.91E-09 | 6.07E-07 | 10.5262928 |
| Zyx | 1.62175759 | 8.2360799 | 11.0660567 | 8.34E-09 | 6.33E-07 | 10.3764716 |
| Gm8818 | 3.21864796 | 1.51605073 | 11.0198082 | 8.84E-09 | 6.65E-07 | 10.5469281 |
| Ccnd3 | 1.5478576 | 7.65806898 | 11.017863 | 8.87E-09 | 6.65E-07 | 10.2971441 |
| Gbp7 | 1.62813918 | 7.95329967 | 10.8756099 | 1.06E-08 | 7.89E-07 | 10.117991 |
| Rab11fip4 | 1.62105125 | 6.20768803 | 10.8283498 | 1.13E-08 | 8.34E-07 | 10.0321098 |
| Rxra | 2.37059352 | 2.83655458 | 10.7829924 | 1.20E-08 | 8.79E-07 | 10.220406 |
| Klrg1 | 4.94500112 | 1.7453238 | 10.7690739 | 1.22E-08 | 8.90E-07 | 10.2389434 |
| Pea15a | 1.83382064 | 6.80231456 | 10.6857628 | 1.36E-08 | 9.76E-07 | 9.83969379 |
| Ly6c2 | 1.71765452 | 6.57934924 | 10.6615462 | 1.40E-08 | 1.00E-06 | 9.80604623 |
| Snx9 | 3.61740626 | 0.52666925 | 10.6492949 | 1.42E-08 | 1.01E-06 | 10.0021028 |
| Car5b | 1.71920874 | 4.65201192 | 10.6340526 | 1.45E-08 | 1.02E-06 | 9.85844117 |
| Lamc1 | 3.20591411 | 1.32113242 | 10.6250011 | 1.47E-08 | 1.03E-06 | 10.046725 |
| Cd38 | 1.59022386 | 4.76581943 | 10.6087905 | 1.50E-08 | 1.05E-06 | 9.81462268 |
| Ctsd | 1.70140575 | 9.81492049 | 10.5932456 | 1.53E-08 | 1.06E-06 | 9.79366479 |
| Rrm2 | 1.58636617 | 4.8318105 | 10.533385 | 1.66E-08 | 1.13E-06 | 9.70722197 |
| Mxd1 | 1.63760339 | 4.83635574 | 10.5036373 | 1.72E-08 | 1.17E-06 | 9.66651061 |
| Adgre5 | 1.44680293 | 8.53755382 | 10.5015373 | 1.73E-08 | 1.17E-06 | 9.63313407 |
| Ifitm10 | 1.74830658 | 4.64325378 | 10.4831672 | 1.77E-08 | 1.19E-06 | 9.65549839 |
| Ptprv | 2.13133601 | 4.18809862 | 10.4405811 | 1.87E-08 | 1.24E-06 | 9.6403602 |

| | | | | | | |
|---|---|---|---|---|---|---|
| Adam19 | 1.72708458 | 6.40806364 | 10.4389488 | 1.87E-08 | 1.24E-06 | 9.50424373 |
| Socs2 | 3.70829629 | 1.16059287 | 10.3093718 | 2.23E-08 | 1.43E-06 | 9.6367016 |
| Sdcbp2 | 1.5249421 | 7.02834811 | 10.3084703 | 2.23E-08 | 1.43E-06 | 9.3280418 |
| Tmem154 | 1.35612508 | 6.43495579 | 10.2575671 | 2.39E-08 | 1.52E-06 | 9.25396104 |
| Icam1 | 1.39177897 | 6.50777695 | 10.2091924 | 2.55E-08 | 1.60E-06 | 9.18634386 |
| Ccr8 | 2.35478347 | 2.48911133 | 10.1620355 | 2.71E-08 | 1.67E-06 | 9.41994798 |
| Ildr1 | 1.66738688 | 4.4205191 | 10.143168 | 2.78E-08 | 1.68E-06 | 9.20893167 |
| Abi3 | 1.916699 | 5.02808665 | 10.1306898 | 2.83E-08 | 1.70E-06 | 9.13714479 |
| Ranbp10 | 1.48243416 | 5.04071783 | 10.1170202 | 2.88E-08 | 1.73E-06 | 9.11702792 |
| Plekhg3 | 4.09439108 | 0.75996467 | 10.1098293 | 2.91E-08 | 1.74E-06 | 9.35317654 |
| Mcoln2 | 1.69731968 | 4.57349816 | 10.0669969 | 3.09E-08 | 1.82E-06 | 9.08794653 |
| Il12rb1 | 1.52121892 | 5.17466414 | 10.0318099 | 3.24E-08 | 1.91E-06 | 8.9872116 |
| Ifitm3 | 2.06822463 | 3.29981446 | 10.0256963 | 3.26E-08 | 1.91E-06 | 9.16252579 |
| Hopx | 1.56494859 | 4.66259242 | 9.98830902 | 3.44E-08 | 2.01E-06 | 8.96899547 |
| Bcl2l1 | 1.36131885 | 6.70052957 | 9.95865 | 3.58E-08 | 2.08E-06 | 8.8341646 |
| Slamf7 | 1.36446995 | 5.46535279 | 9.94925401 | 3.62E-08 | 2.09E-06 | 8.85109259 |
| Selplg | 1.5905908 | 10.0312646 | 9.94714884 | 3.63E-08 | 2.09E-06 | 8.90776429 |
| Rexo5 | 2.04822137 | 3.76135919 | 9.87152272 | 4.03E-08 | 2.30E-06 | 8.89639882 |
| Zfp652 | 1.60995923 | 4.35211005 | 9.81705617 | 4.35E-08 | 2.46E-06 | 8.75634364 |
| Ccrl2 | 2.17295319 | 2.59972518 | 9.76776834 | 4.65E-08 | 2.60E-06 | 8.86849348 |
| Lair1 | 2.01146762 | 3.53007623 | 9.7674206 | 4.66E-08 | 2.60E-06 | 8.77470353 |
| Dusp5 | 1.72282003 | 3.99619273 | 9.76603082 | 4.67E-08 | 2.60E-06 | 8.72087326 |
| Rcbtb2 | 1.33266803 | 6.53674914 | 9.74304049 | 4.82E-08 | 2.65E-06 | 8.52512234 |
| Gbp4 | 1.42512312 | 7.02466253 | 9.73839628 | 4.85E-08 | 2.65E-06 | 8.522451 |
| Sla2 | 1.3776351 | 6.44677381 | 9.72555098 | 4.94E-08 | 2.69E-06 | 8.5002064 |
| Zbtb38 | 1.33368153 | 7.11766445 | 9.6737793 | 5.31E-08 | 2.85E-06 | 8.43057954 |
| Nedd4 | 3.1604269 | 0.87563883 | 9.65223632 | 5.47E-08 | 2.93E-06 | 8.75553863 |
| Lrrc8c | 1.29133067 | 6.82420011 | 9.61947444 | 5.73E-08 | 3.04E-06 | 8.34725243 |
| Plxdc1 | 2.62899486 | 3.00937964 | 9.60465264 | 5.85E-08 | 3.10E-06 | 8.59946964 |
| Snai3 | 2.1697131 | 3.01946256 | 9.59362147 | 5.94E-08 | 3.13E-06 | 8.58264077 |
| Peak1 | 1.6076486 | 4.39650419 | 9.59180277 | 5.95E-08 | 3.13E-06 | 8.42775649 |
| AA467197 | 3.19670827 | 0.5719742 | 9.56936662 | 6.14E-08 | 3.22E-06 | 8.62471278 |
| Gm33104 | 3.56864462 | 0.06774774 | 9.5091285 | 6.69E-08 | 3.46E-06 | 8.4861011 |
| Cd200r4 | 2.9166401 | 1.14251758 | 9.50042264 | 6.77E-08 | 3.49E-06 | 8.55475537 |
| Ehd1 | 1.33582648 | 5.54683121 | 9.47475293 | 7.02E-08 | 3.61E-06 | 8.1605501 |
| Ppp1r3b | 3.10794533 | 0.93463474 | 9.44798481 | 7.30E-08 | 3.73E-06 | 8.47855455 |
| Nccrp1 | 3.81140746 | -0.0985932 | 9.43753973 | 7.41E-08 | 3.77E-06 | 8.36494639 |
| Lrrn4 | 4.20169257 | 1.13294329 | 9.4271785 | 7.52E-08 | 3.82E-06 | 8.45300309 |
| Arl5a | 1.46125335 | 5.32186457 | 9.39180266 | 7.91E-08 | 3.98E-06 | 8.05231597 |
| Cdk6 | 1.4602242 | 7.32295825 | 9.37164584 | 8.14E-08 | 4.09E-06 | 7.99155084 |
| Ltb4r1 | 3.97912143 | 0.27855556 | 9.35509999 | 8.33E-08 | 4.17E-06 | 8.30769141 |
| Runx3 | 1.29043763 | 6.52088719 | 9.33851629 | 8.53E-08 | 4.24E-06 | 7.93203982 |

| | | | | | | |
|---|---|---|---|---|---|---|
| Trex1 | 1.44893298 | 5.8317959 | 9.31430205 | 8.83E-08 | 4.37E-06 | 7.90918595 |
| Gzmc | 3.14942775 | 0.82090464 | 9.29160857 | 9.13E-08 | 4.50E-06 | 8.25818927 |
| Crybg1 | 1.30915189 | 5.69904331 | 9.23805283 | 9.86E-08 | 4.79E-06 | 7.80092862 |
| Alpk2 | 3.30779633 | 0.65964244 | 9.23669756 | 9.88E-08 | 4.79E-06 | 8.17509153 |
| Apbb1 | 1.76742466 | 4.19166459 | 9.1731394 | 1.08E-07 | 5.19E-06 | 7.83229002 |
| Sord | 1.5079561 | 4.34231327 | 9.16621684 | 1.09E-07 | 5.21E-06 | 7.80540318 |
| St6galnac1 | 1.26424391 | 7.18757645 | 9.09124913 | 1.22E-07 | 5.73E-06 | 7.56757395 |
| Pitpnc1 | 1.3245096 | 7.9755474 | 9.01388717 | 1.37E-07 | 6.29E-06 | 7.47235715 |
| Cd86 | 2.43428728 | 2.63465 | 9.00356487 | 1.39E-07 | 6.34E-06 | 7.75976484 |
| Srebf2 | 1.24964124 | 6.69252382 | 9.00113646 | 1.39E-07 | 6.34E-06 | 7.42329893 |
| Slc2a3 | 1.86471357 | 5.24333794 | 8.99460707 | 1.41E-07 | 6.38E-06 | 7.46079232 |
| Itgae | 2.86049028 | 1.12398225 | 8.99123451 | 1.41E-07 | 6.39E-06 | 7.83433713 |
| Podnl1 | 1.26682102 | 8.57629266 | 8.98845886 | 1.42E-07 | 6.40E-06 | 7.45330578 |
| Ccr9 | 3.79379864 | 0.21636623 | 8.98164939 | 1.43E-07 | 6.43E-06 | 7.78788319 |
| Misp3 | 2.45462653 | 2.20224892 | 8.98090314 | 1.44E-07 | 6.43E-06 | 7.76567878 |
| Cela1 | 1.91214433 | 3.22817308 | 8.96681605 | 1.47E-07 | 6.53E-06 | 7.63756046 |
| Nxpe4 | 3.53606978 | 0.09491598 | 8.96119218 | 1.48E-07 | 6.55E-06 | 7.74713955 |
| Prr5l | 1.69189572 | 4.12643371 | 8.94872894 | 1.51E-07 | 6.65E-06 | 7.50061121 |
| Racgap1 | 1.30572327 | 5.65070638 | 8.91747986 | 1.58E-07 | 6.92E-06 | 7.3165518 |
| Gm20696 | 2.53198916 | 1.45075843 | 8.91215108 | 1.59E-07 | 6.94E-06 | 7.71038455 |
| Capn2 | 1.29466956 | 6.70866127 | 8.91157521 | 1.59E-07 | 6.94E-06 | 7.28600246 |
| Rhoc | 1.95057062 | 3.04430405 | 8.87039094 | 1.69E-07 | 7.33E-06 | 7.51382039 |
| Serpini1 | 1.59717548 | 3.90806752 | 8.86253147 | 1.71E-07 | 7.39E-06 | 7.39459342 |
| Ptpn4 | 1.82829389 | 3.78728731 | 8.85909516 | 1.72E-07 | 7.41E-06 | 7.4040317 |
| Tapbpl | 1.28742457 | 7.41959106 | 8.84648365 | 1.75E-07 | 7.53E-06 | 7.19820324 |
| Gm47586 | 3.26027948 | 2.56524663 | 8.81887419 | 1.83E-07 | 7.77E-06 | 7.48918166 |
| AU020206 | 1.24896849 | 7.63687516 | 8.81176991 | 1.85E-07 | 7.83E-06 | 7.15070255 |
| Atf6 | 1.3801587 | 4.35835179 | 8.749243 | 2.03E-07 | 8.52E-06 | 7.16768901 |
| Usp20 | 1.59911839 | 4.20295418 | 8.74643516 | 2.04E-07 | 8.53E-06 | 7.18095742 |
| Lztfl1 | 1.51318445 | 3.72647883 | 8.72327813 | 2.11E-07 | 8.80E-06 | 7.2026268 |
| Fam20a | 1.61775108 | 3.75272469 | 8.71754909 | 2.13E-07 | 8.85E-06 | 7.19044235 |
| Anxa4 | 1.42758062 | 4.42934033 | 8.70122534 | 2.18E-07 | 8.99E-06 | 7.0852812 |
| Itih5 | 2.04905098 | 2.90977882 | 8.69090392 | 2.21E-07 | 9.10E-06 | 7.25568787 |

**Supplemental Table 2.** The top 200 highly expressed genes in cluster-3 cells were derived from bulk RNAseq data in **Fig.3 d.**
