# Supplementary material for: Neoantigen-specific cytotoxic Tr1 CD4 T cells suppress cancer immunotherapy
Source: Nature. 2024 Jul 24;632(8023):182–91. doi: 10.1038/s41586-024-07752-y (PMC11291290; doi:10.1038/s41586-024-07752-y)
Supplement: Supplementary file 2 — Reporting Summary [file 41586_2024_7752_MOESM2_ESM.pdf]

nature portfolio

Corresponding author(s): Robert Schreiber

Last updated by author(s): May 22, 2024

Reporting Summary

Nature Portfolio wishes to improve the reproducibility of the work that we publish. This form provides structure for consistency and transparency in reporting. For further information on Nature Portfolio policies, see our Editorial Policies and the Editorial Policy Checklist.

Statistics

For all statistical analyses, confirm that the following items are present in the figure legend, table legend, main text, or Methods section.

n/a	Confirmed	
☐	☒	The exact sample size (*n*) for each experimental group/condition, given as a discrete number and unit of measurement
☐	☒	A statement on whether measurements were taken from distinct samples or whether the same sample was measured repeatedly
☐	☒	The statistical test(s) used AND whether they are one- or two-sided<br>*Only common tests should be described solely by name; describe more complex techniques in the Methods section.*
☒	☐	A description of all covariates tested
☐	☒	A description of any assumptions or corrections, such as tests of normality and adjustment for multiple comparisons
☐	☒	A full description of the statistical parameters including central tendency (e.g. means) or other basic estimates (e.g. regression coefficient) AND variation (e.g. standard deviation) or associated estimates of uncertainty (e.g. confidence intervals)
☐	☒	For null hypothesis testing, the test statistic (e.g. *F*, *t*, *r*) with confidence intervals, effect sizes, degrees of freedom and *P* value noted<br>*Give P values as exact values whenever suitable.*
☒	☐	For Bayesian analysis, information on the choice of priors and Markov chain Monte Carlo settings
☒	☐	For hierarchical and complex designs, identification of the appropriate level for tests and full reporting of outcomes
☒	☐	Estimates of effect sizes (e.g. Cohen's *d*, Pearson's *r*), indicating how they were calculated

Our web collection on statistics for biologists contains articles on many of the points above.

Software and code

Policy information about availability of computer code

Data collection	Flow cytomtery data was collected using BD FACSDIVA software V9.1. MultiPlex assay was performed using Flex MAP 3D. ELISPOT was collected using a CTL Immunospot S6 universal machine and software.
Data analysis	All statistical analysis was performed using GraphPad Prism software version 10.2.2. cell ranger v6.1.1 was used for scRNAseq

For manuscripts utilizing custom algorithms or software that are central to the research but not yet described in published literature, software must be made available to editors and reviewers. We strongly encourage code deposition in a community repository (e.g. GitHub). See the Nature Portfolio guidelines for submitting code & software for further information.

Data

Policy information about availability of data

All manuscripts must include a data availability statement. This statement should provide the following information, where applicable:

- Accession codes, unique identifiers, or web links for publicly available datasets
- A description of any restrictions on data availability
- For clinical datasets or third party data, please ensure that the statement adheres to our policy

All data supporting the findings of this study are available within the paper, its Supplemental or Extended Data files, or Source files. Any additional information related to the study is available from the corresponding author upon request. All reagents are available through a Material Transfer Agreement.

Research involving human participants, their data, or biological material

Policy information about studies with human participants or human data. See also policy information about sex, gender (identity/presentation), and sexual orientation and race, ethnicity and racism.

Reporting on sex and gender	N/A
Reporting on race, ethnicity, or other socially relevant groupings	N/A
Population characteristics	N/A
Recruitment	N/A
Ethics oversight	N/A

Note that full information on the approval of the study protocol must also be provided in the manuscript.

Field-specific reporting

Please select the one below that is the best fit for your research. If you are not sure, read the appropriate sections before making your selection.

☒ Life sciences ☐ Behavioural & social sciences ☐ Ecological, evolutionary & environmental sciences

For a reference copy of the document with all sections, see nature.com/documents/nr-reporting-summary-flat.pdf

Life sciences study design

All studies must disclose on these points even when the disclosure is negative.

Sample size	Sample sizes were determined based on 25 years experience with these types of tumors in general, and over 10 years with T3 and F244 cell lines. (Gubin, et al 2014, Alspach, 2019)
Data exclusions	No data were excluded
Replication	All experiments had multiple biological and/or technical replicates and are indicated the Figure legends.
Randomization	For animal studies, mice were randomly assigned from large batches obtained from the vendor to different experimental groups in an age-matched distribution.
Blinding	Tumor injection was performed by one person. Treatments and tumor measurement were performed by independent people. No blinding was performed in vitro studies.

Reporting for specific materials, systems and methods

We require information from authors about some types of materials, experimental systems and methods used in many studies. Here, indicate whether each material, system or method listed is relevant to your study. If you are not sure if a list item applies to your research, read the appropriate section before selecting a response.

Materials & experimental systems

n/a	Involved in the study
☐	☒ Antibodies
☐	☒ Eukaryotic cell lines
☒	☐ Palaeontology and archaeology
☐	☒ Animals and other organisms
☒	☐ Clinical data
☒	☐ Dual use research of concern
☒	☐ Plants

Methods

n/a	Involved in the study
☒	☐ ChIP-seq
☐	☒ Flow cytometry
☒	☐ MRI-based neuroimaging

Antibodies

Antibodies used

Flow antibodies were purchased from Antibodies were purchased from: [BioLegend; IFN (XMG1.2; 1:100; cat# 505808), TNF-(XMG1.2; 1:100; cat# 505826), CD200 (OX-90; 1:200; 123820), PD-1 (29F.1A12; 1:200; cat# 135228), CCL5 (2E9/CCL5; 1:500; cat# 149106), GZMB (QA16A02; 1:50; cat# 372216), TIM-3 (RMT3-23; 1:200; cat# 119723), CD25 (PC61; 1:100; cat# 102036), CD154 (MR1;1:100; cat# 106506), CD152 (UC10-4B9; 1:100; cat# 106318), CD4 (RM4-5: 1:500), IL-2 (Jes6-5H4: 1:50; cat# 503826), LILRB4 (H1.1: 1:200; cat# 144904), CD11b (M1/70: 1:800; cat# 101226), XCR1 (ZET: 1:100; cat# 148206), MHC-II (M5/114.15.2: 1:1000; cat# 107641), CD11c (N418: 1:200; cat# 117336), CD172a (P84: 1:500; cat# 144008), (Zombie NIR fixable viability dye; 1:500; cat# 423106), CD86 (GL-1; 1:200; cat# 105042), CD80 (16-10A1; 1:200; cat# 104712), CD40 (FGK45; 1:100; cat# 157506);

BD Bioscience: CD39 (Y23-1185; 1:400; cat# 567105), CD153 (RM153; 1:200; cat# 740751), CD70 (FR70; 1:100; cat# 740741) and CD8 (53.6.7; 1:200; cat# 564920); or

eBioSciences: FOXP3 (Fjk-16a; 1:50; cat# 11-5773-82) and SEMA4a (5E3; 1:50; cat# 46-9753-41)]. Foxp3/Transcription factor Staining kit (ThermoFischer; cat# 00-5523-00) was used to stain Foxp3 and other intracellular proteins according to the manufacturing protocol.

Validation

All primary antibodies were validated against indicated proteins by the manufacturer per their associated Data Sheets and are included on their websites.

Eukaryotic cell lines

Policy information about cell lines and Sex and Gender in Research

Cell line source(s)

T3, F244 and 1956 cell lines were T3, F244 and 1956 cell lines were generated via subcutaneous injection of MCAnerated via subcutaneous injection of MCA. HEK293 cell line were obtained from ATCC (CRL-1573).

Authentication

Cell lines were authenticated by whole exosome sequencing and expression of certain genes

Mycoplasma contamination

All cell lines are routinely tested each month and were negative for mycoplasma.

Commonly misidentified lines
(See ICLAC register)

This study did not involve any commonly misidentified cell lines.

Animals and other research organisms

Policy information about studies involving animals; ARRIVE guidelines recommended for reporting animal research, and Sex and Gender in Research

Laboratory animals

Six-8-week-old female C57BL/6J mice (Cat # 000664, Jackson Laboratory) , GZMB KO, LILRB4 KO, Rag2-deficient and wild type male 129S6 mice were used for experiments. Mice were housed in groups of 3 to 5. Photoperiod = 12 hr on:12 hr off dark/light cycle. Ambient animal room temperature is 70° F, controlled within ±2° and room humidity is 50%, controlled within ±5%.

Wild animals

No wild animals were used in this study.

Reporting on sex

Both male mice (129S6) were used for T3 and F244 tumors and female mice (C57BL6) were used for 1956 tumor line.

Field-collected samples

No field collected samples were used in this study.

Ethics oversight

All animal studies were performed with the approval of the Association for the Accreditation of Laboratory Animal Care-accredited Animal Studies Committee of Washington University in St. Louis

Note that full information on the approval of the study protocol must also be provided in the manuscript.

Plants

Seed stocks	N/A
Novel plant genotypes	N/A
Authentication	N/A

Flow Cytometry

Plots

Confirm that:

- [x] The axis labels state the marker and fluorochrome used (e.g. CD4-FITC).
- [x] The axis scales are clearly visible. Include numbers along axes only for bottom left plot of group (a 'group' is an analysis of identical markers).
- [x] All plots are contour plots with outliers or pseudocolor plots.
- [x] A numerical value for number of cells or percentage (with statistics) is provided.

Methodology

Sample preparation	Tumours were harvested, manually disassociated into small pieces and digested with collagenase 1A for 45 minutes at 37 c. Single cell suspension was then filter through 70 um cell strainer. Subsequently, Red blood cells were lysed. Suspension were filtered again through 40 um cell strainer and resuspended into a complete RPMI media. For intracellular stains, cells were fixed and permeabilized using the BD Foxp3 staining kit
Instrument	BD FACS Symphony 3, LSRFortessaX20 were used to collect the data. ARIA-II and/or Bigfoot were used for sorting.
Software	BD FACSDIVA software V9.1 was used for collecting the flow cytometry data and for sorting, Data were analyzed using FlowJo software version 10.10.
Cell population abundance	Cell abundance was shown through out gating strategies
Gating strategy	Live Lymphocytes gated using CD90.2 and CD4. Antigen-specific cells were gated using the appropriate tetramer,Tregs were excluded using CD25 and Foxp3 staining. Expressions of CD39, CD200 CD153, LILRB4, GZMB or CCL5 were measured.

- [x] Tick this box to confirm that a figure exemplifying the gating strategy is provided in the Supplementary Information.
